# Chronic Inflammation Might Protect Hemodialysis Patients From Severe COVID-19

**DOI:** 10.3389/fimmu.2022.821818

**Published:** 2022-02-21

**Authors:** Barbara Prietl, Balazs Odler, Alexander H. Kirsch, Katharina Artinger, Manfred Eigner, Sabine Schmaldienst, Verena Pfeifer, Stefanie Stanzer, Anita Eberl, Reingard Raml, Thomas Pieber, Alexander R. Rosenkranz, Marianne Brodmann, Philipp Eller, Kathrin Eller

**Affiliations:** ^1^ Center for Biomarker Research in Medicine, Graz, Austria; ^2^ Division of Endocrinology and Diabetology, Department of Internal Medicine, Medical University of Graz, Graz, Austria; ^3^ Division of Nephrology, Department of Internal Medicine, Medical University of Graz, Graz, Austria; ^4^ Klinikum Favoriten, Wiener Krankenanstaltenverbund, Vienna, Austria; ^5^ Institute for Biomedicine and Health Sciences, Joanneum Research Forschungsgesellschaft mbH, Graz, Austria; ^6^ Division of Angiology, Department of Internal Medicine, Medical University of Graz, Graz, Austria; ^7^ Intensive Care Unit, Department of Internal Medicine, Medical University of Graz, Graz, Austria

**Keywords:** inflammation, COVID-19, hemodialysis patients, CD8^+^ T cells, cytokines

## Abstract

Hemodialysis patients (HD) are expected to have excess mortality in coronavirus disease 2019 (COVID-19). This was challenged by a recent study reporting HD patients to have comparable mortality and less ICU admissions when hospitalized with COVID-19. An altered immune system due to chronic inflammation might protect HD-patients from severe COVID-19. Therefore, we aimed to describe the peripheral blood immune phenotype in HD-patients and respective controls with COVID-19.

**Methods:**

Sixty-four patients (31 HD, 33 non-HD) with PCR-confirmed COVID-19 and 16 control patients (10 HD, 6 non-HD) were prospectively included. According to symptoms, COVID-19 patients were categorized as asymptomatic/mild, moderate or severe COVID-19 phenotypes. Cytokine profiling and immune phenotyping was performed.

**Results:**

Th1 and Th17 plasma cytokine levels were highly increased in HD patients without COVID-19 and were not significantly regulated during COVID-19. In non-HD COVID-19 patients these cytokines increased significantly with disease severity. While all patients with moderate or severe COVID-19 showed hallmarks of COVID-19 such as decreased CD3^+^, CD4^+^ and CD8^+^ and CD4^+^CD25^hi^FoxP3^+^ regulatory T cells, significantly increased CD38^+^CD8^+^ effector memory and CD38^+^CD8^+^ TEMRA T cells were detected in moderate/severe COVID-19 HD patients, which was not observed in non-HD patients with moderate or severe COVID-19. Furthermore, CD161^+^CD8^+^ T cells decreased significantly in non-HD COVID-19 patients dependent on disease severity, but not in HD patients. Dynamics of B cells and subtypes were comparable in HD and non-HD COVID-19 patients.

**Conclusions:**

HD patients might be protected from severe COVID-19 due to their chronic inflammatory state with increased CD38^+^CD8^+^ effector memory and TEMRA T cells as well as CD161^+^CD8^+^ T cells.

## Introduction

The coronavirus disease 2019 (COVID-19) pandemic induced by severe acute respiratory syndrome coronavirus 2 (SARS-CoV-2) is a world-wide crisis and has increased mortality in patients with underlying chronic diseases such as diabetes, obesity, coronary heart disease, hypertension and chronic kidney disease (CKD) (1). Patients with end-stage renal disease and the need for renal replacement therapy suffer from multiple comorbidities and might thus be expected to be a very vulnerable cohort with high mortality due to COVID-19. However, largely different outcomes have been reported concerning mortality in the hemodialysis (HD) population in the last years. Xiong et al. reported that HD-patients experienced fewer symptoms and required less intensive care treatment than expected in a Chinese cohort (2). In contrast, an observational study from the U.S. showed significantly higher mortality rates in HD-patients but lacked a comparator group (3). There are two register analyses from the US and one from the ERA-EDTA showing a mortality rate in HD-patients ranging between 20 to 31%, respectively (4, 5). These data have been challenged by a recent retrospective cohort study comparing patients with HD-therapy hospitalized due to COVID-19, who were propensity matched to patients without kidney failure (6). They reported significantly decreased in-hospital mortality rates of 10%, which did not differ from patients without HD. Interestingly, HD-patients were significantly less likely to be admitted to ICU, had less need for mechanical ventilation and experienced overall less severe COVID-19 symptoms as compared to matched controls (6). There have been various speculations that uremia might chronically tamper the immune system and might protect patients from hyperinflammation and cytokine storm that is supposed to lead to pulmonary failure in COVID-19.

The immune system of HD-patients is critically altered. While the innate immune system has been described to be activated, the adaptive immune system is severely impaired (7). It is generally accepted that uremic toxins activate innate immune cells to produce increased reactive oxygen species as well as pro-inflammatory cytokines, which critically decrease T and B cell numbers as well as their function (7). Furthermore, pre-mature aging of adaptive immune cells has been described in HD-patients (8). Together, these alterations in the immune system make HD-patients more susceptible to infections, cardiovascular events and malignancies (7).

The immune reaction in COVID-19 has been intensively studied since the start of the pandemic. The innate immune response is critical for the first phase of disease to restrict viral replication within infected cells, to recruit innate immune cells and to prime the adaptive immune response. For final elimination of the virus, antibodies produced by effector B cells known as plasma cells and plasmablasts, effector CD4 and CD8 T cells are crucially necessary. Finally, both change into a memory phenotype which helps to fight recurrent infections (9). It is believed that the early innate immune response needs a strong IFN signature to effectively suppress COVID-19. This assumption is supported by the fact that impaired IFN responses are associated with a high risk for fatal COVID-19 (10–12). If the innate response is too slow, the virus starts replicating in the upper and lower respiratory tract and fails to prime adaptive immune responses. Due to the lack of an appropriate T cell response, the innate immune system attempts to control the virus by an overwhelming innate immune response, which results in excessive lung immune infiltration leading to fatal outcomes of COVID-19 (9, 13–15).

The aim of our study was to immune phenotype HD-patients with asymptomatic/mild and moderate/severe COVID-19 and to compare them to non-HD patients in order to gain insights into the peculiar immune response taking place in patients with chronic kidney disease stage 5 (CKD G5) when suffering from COVID-19.

## Material and Methods

### Patient Recruitment and Study Cohort Definitions

Patients were recruited at the Medical University of Graz and the Hospital Favoriten, Vienna, Austria. Eligibility criteria included age ≥18 years and a positive SARS-CoV-2 nasopharyngeal swab tested by RT-PCR. To build the study cohort, all consecutive SARS-CoV2 positive HD patients referred to the study sites between 1^st^ April 2020 and 30^th^ November 2020 were prospectively involved in the study without applying any exclusion criteria except for age. HD patients were routinely screened at their dialysis centers and transferred in case of SARS-CoV2 positivity. Additionally, non-HD patients who required hospital admission due to COVID-19 disease were recruited with the same approach. The non-HD patients, who suffered from asymptomatic/mild COVID-19 were health-care workers routinely screened at our hospital and tested positive for SARS-CoV2. Healthy volunteers and HD patients with a negative SARS-CoV-2 nasopharyngeal swab tested by RT-PCR served as controls. Demographic and clinical data as well as data on patient comorbidities were collected from the patients’ charts. COVID-19 disease was classified according to severity. Asymptomatic patients were SARS-CoV-2 positive on nasopharyngeal swab tested by RT-PCR and did not show symptoms. Mild disease was characterized by symptoms of COVID-19 disease without the need for inpatient treatment. Patients with moderate COVID-19 phenotype required admission to the hospital because of COVID-19. Severe disease phenotype was defined by severe acute respiratory distress syndrome (ARDS) with the need for intensive care treatment and mechanical ventilation. Blood samples were taken immediately after diagnosis and/or admission to the hospital, prior to a scheduled HD session in patients treated with HD. All HD patients were treated with conventional HD using a high-flux membrane.

The study protocol was approved by the Institutional Review Board of the Medical University of Graz, Austria (32-323 ex19/20). All patients gave their informed consent for the study.

### Acute Phase Protein, Cytokine and Chemokine Measurements

For the quantification of acute phase proteins, plasma cytokines and chemokines an immunoassay with electroluminescence detection was performed. The V-Plex Plus Human Biomarker 54-Plex Kit (Mesoscale discovery, Maryland, USA, ref# K15248G-1) was carried out according to the manufacturer’s specifications using a MESO QuickPlex SQ120 device (Mesoscale, USA, ref# AI0AA-0).

### Isolation of PBMC and Flow Cytometry Analysis

Peripheral blood mononuclear cells (PBMCs) were isolated from fresh heparinized whole blood samples (BD vacutainer tubes containing lithium heparin; ref# 367880). Whole blood was diluted 1:1 with PBS (ThermoFisher, ref# A1286301) and layered into a tube prefilled with Lymphoprep density gradient media (Stemcell Technologies, ref# 1114547). Density gradient centrifugation was performed (20min, 800 x g, RT) and the PBMC layer was collected and washed with PBS. Viability and cell number was measured by the use of an automated dual fluorescence cell counter (LUNA-FL, Logos Biosystems) prior to multi-parameter staining of 1 x 10^6^ cells per FACS panel. In total 3 panels were stained per PBMC sample (see **Table 1** for antibody panel information) and one tube with unstained cells served as control. All antibodies were purchased from Becton Dickinson or Thermo Fisher (for details refer to supplementary **Table 1**). Surface panel staining was performed using BD Lyse/Fix buffer according to the manufacturer’s instructions (Becton Dickinson, ref# 558049). Intracellular panel staining was performed using Fix Perm buffer kits (Becton Dickinson, ref# 554714) combined with surface and viability staining (FVS, Becton Dickinson, ref# 564997) prior to permeabilization. Additionally, 50µl fresh whole blood were stained with anti-CD45 APC-H7 antibodies (Becton Dickinson, ref# 560178) and 123count eBeads (Thermo Fisher, ref# 01123442) were added for the analysis of absolute numbers of leukocyte subpopulations. All samples were acquired on a four-laser BD FACS Fortessa SORP instrument. UltraComp eBeads (ThermoFisher, ref# 01222242) were used for compensation and FMO controls were applied for appropriate gating of T cell and B cell subtypes.

**Table 1:** FACS panels used to characterize T and B cells in peripheral blood.

Panel 1		Panel 2		Panel 3	
Marker	Fluorochrome	Marker	Fluorochrome	Marker	Fluorochrome
CD3	APC-H7	CD3	AF700	CD19	PE
CD4	BV605	CD4	PE-CF594	CD20	APC-H7
CD45RA	BV786	CD8	BV711	IgD	PerCp-Cy5.5
CD15S	BV421	CD45RA	BV786	IgM	BB515
Ki-67	AF488	CD27	APC-H7	CD24	BV711
FoxP3	PE	CD57	FITC	CD27	BV786
CD8	BV711	CD279	PE	CD86	PE-CF594
CD161	APC	CD197	AF647	CD5	PE-Cy7
CD127	BV510	HLA-DR	BV650	CD38	APC-R700
FVS	APC-R700	CD127	PerCpCy5.5	CD10	BV510
CD25	PE-Cy7	CD95	BV605	CD21	BV421
CD39	PE-CF594	CD38	BV421	CCR7	AF647
CD147	PerCP-Cy5.5	CD25	PE-Cy7		
		CD28	BV510		

### Statistical Analysis

Prior to data analysis, all metric outcome variables were tested for normality by using the Shapiro-Wilk test. In accordance with the result, the one-way ANOVA test or the Kruskal-Wallis test was applied to the data and Tukey’s multiple comparison test or Dunn multiple comparison test was used for final *post hoc* analysis of differences. Statistical evaluation was performed using GraphPad Prism 9 (San Diego, CA, USA).

## Results

### Study Population

We recruited 64 patients with SARS-CoV-2 positive PCR for this study. Thereof, thirty-one patients were dependent on HD. Eighteen and nine patients were included with asymptomatic or mild SARS-CoV-2 infection in the HD and non-HD population, respectively. Thirteen HD patients (11 moderate, 2 severe) and 24 patients (11 moderate, 13 severe) without HD dependence were recruited with moderate and severe COVID-19, respectively. Ten HD patients without SARS-CoV-2 infection and six healthy controls were included as controls. Patient characteristics are listed in **Table 2**. In our patient cohort, no significant difference in mortality rates due to SARS-CoV-2 infection within the first month after infection was detected between non-HD and HD patients (4/31 [12.9%] HD *vs* 6/33 non-HD [18.2%] patients). Significantly fewer HD patients were admitted to ICU treatment compared to non-HD patients (1/13 [7.7%] HD *vs*. 12/24 [50%] non-HD patients) in patients with moderate/severe COVID-19 disease. No significant differences between steroid and remdesivir treatment of moderate and severe COVID-19 non-HD compared to moderate/severe COVID-19 HD patients was observed (**Table 2**; p=0.558 for steroids and p=0.327 for remdesivir). Significantly more severe COVID-19 non-HD patients received convalescent plasma (**Table 2**; p=0.021). Of note, significantly more HD patients suffered from hypertension compared to non-HD patients, while no significant differences were observed in the comorbidities such as diabetes and heart disease (**Table 2**). Since only 2 HD patients with severe phenotype were recruited to the study, we were not able to evaluate them separately due to statistical reasons and thus analyzed them together with the moderate phenotype HD patients.

**Table 2:** Clinical characteristics of the study participants without and with SARS-CoV-2 infection.

	Patients not on hemodialysis					Patients on hemodialysis			p-value
	Negative N = 6	Asympt./mild N = 9		Moderate N = 11	Severe N = 13	Negative N = 10	Asympt./mild N = 18	Moderate/severe N = 13	
**Age (years)**	41 (34-46)	65 (40-92)		60 (40-90)	70 (36-88)	63 (34-85)	73 (22-93)	69 (34-83)	0.062
**BMI (kg/m^2^)**	–	–		27.0 (22.5-38.4)	25.7 (17.7-32.8)	24.8 (18.6-31.3)	26.1 (20.1-36.5)	25.5 (18.6-33.8)	0.885
**Sex (male)**	5 (83)	3 (33)		6 (55)	8 (62)	8 (80)	11 (61)	6 (46)	0.351
**Ethnicity**									
White	6 (100)	9 (100)		10 (91)	13 (100)	10 (100)	18 (100)	12 (92)	0.573
Black	0 (0)	0 (0)		1 (9)	0(0)	0 (0)	0 (0)	1 (8)	0.573
**Comorbidities**									
Diabetes mellitus	–	2 (22)		3 (27)	4 (31)	4 (40)	8 (44)	5 (38)	0.926
Arterial hypertension	–	1 (11)		9 (82)	7 (54)	9 (90)	18 (100)	11 (85)	**0.003**
Pulmonary disease	–	1 (11)		3 (27)	4 (31)	1 (10)	2 (11)	2 (15)	0.686
Chronic heart disease	–	3 (33)		4 (36)	5 (38)	7 (70)	10 (56)	9 (69)	0.356
Chronic kidney disease	–	1 (11)		4 (36)	6 (46)	10 (100)	18 (100)	13 (100)	**<0.001**
Dyslipidemia	–	1 (11)		4 (36)	4 (31)	3 (30)	9 (50)	8 (62)	0.503
Systemic autoimmune disease	–	1 (11)		0 (0)	2 (15)	1 (10)	1 (6)	2 (15)	0.645
**Death**	–	0 (0)		0 (0)	6 (46)	0 (0)	1 (6)	3 (23)	**0.002**
**ICU admission**	–	0 (0)		0 (0)	12 (92)	0 (0)	0 (0)	1 (8)	**<0.001**
Catecholamine	–	0 (0)		0 (0)	3 (23)	0 (0)	0 (0)	0 (0)	
CVVH	–	0 (0)		0 (0)	1 (8)	0 (0)	0 (0)	1 (8)	
Ventilation	–	0 (0)		0 (0)	6 (46)	0 (0)	0 (0)	0 (0)	
**Therapies**									
Remdesivir	–	0 (0)		2 (18)	2 (15)	–	0 (0)	0 (0)	0.303
Steroids	–	0 (0)		7 (63)	9 (69)	–	1 (5)	10 (77)	**<0.001**
Convalescent plasma	–	0 (0)		0 (0)	4 (31)	–	0 (0)	0 (0)	**0.014**
Tocilizumab	–	0 (0)		0 (0)	1 (8)	–	0 (0)	0 (0)	0.455

Significances between all groups evaluated by Kruskal-Wallis test are provided. (BMI, body mass index; ICU, intensive care unit; CVVH, continuous veno-venous hemofiltration).

Significances are shown in bold values.

### HD Patients Show Chronic Inflammation in Cytokine Profiling, Which Is Not Regulated During COVID-19

**Figure 1:**
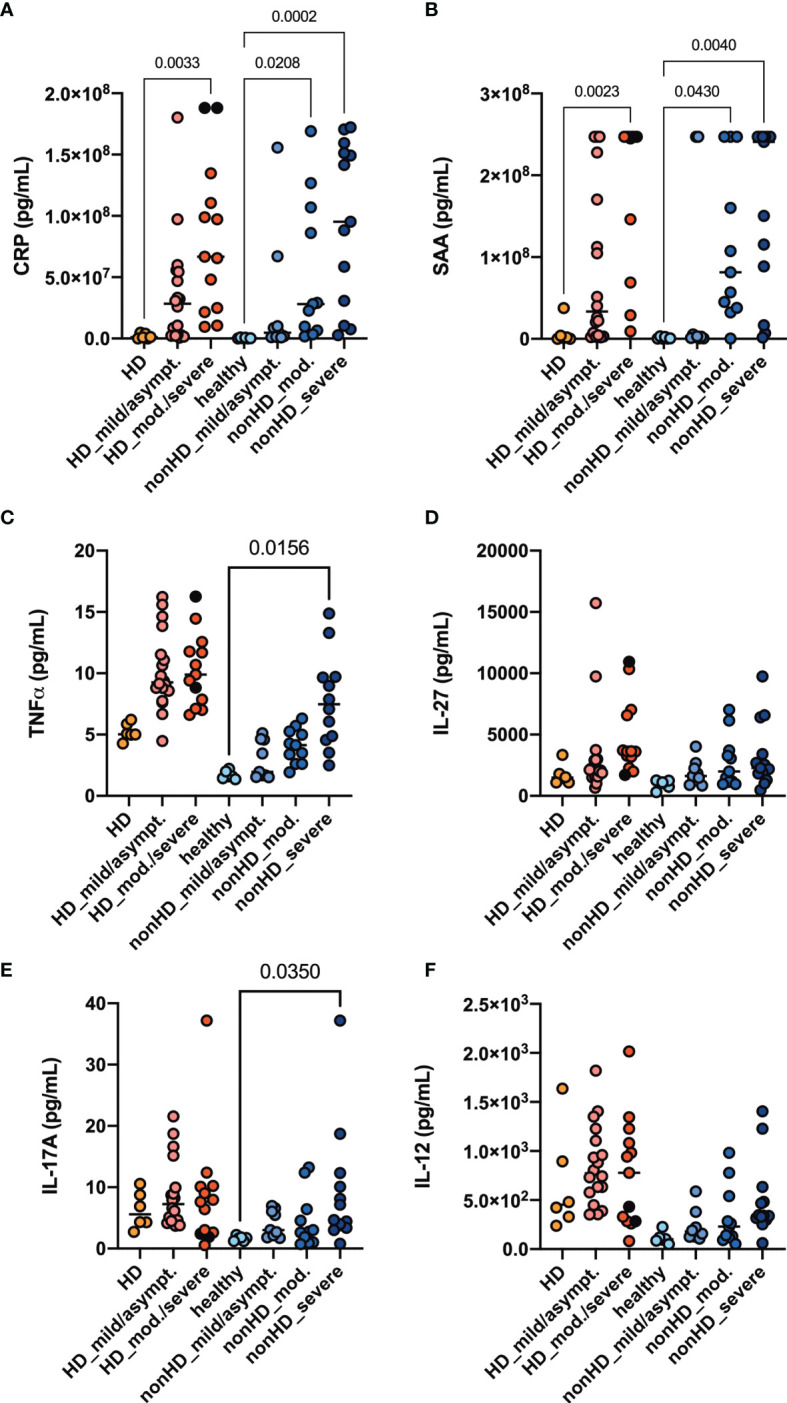
Acute phase parameters, Th1 and Th17 plasma cytokine levels. HD- and non-HD patients without SARS-CoV-2 infection (orange circles, HD: n = 6; light blue circles, non-HD: 6), with mild/asymptomatic (light red circles, HD: n = 18; mid blue circles, non-HD: n = 9), moderate (red circles, HD: n = 11; blue circles, non-HD: n = 11) and severe COVID-19 (black circles, HD: n = 2; dark blue circles, non-HD: n = 13) were included. Severe and moderate COVID-19 HD patients were analyzed as one group. Plasma-levels of the acute phase parameters **(A)** CRP and **(B)** serum amyloid A (SAA) as well as the cytokines **(C)** TNF*α*, **(D)** IL - 27, **(E)** IL - 17A and **(F)** IL - 12 were evaluated. Data are shown as individual values as well as median. Only significances within the HD or non-HD collective are shown.

We performed cytokine profiling by measuring chemokines and acute phase parameters in the plasma of our cohort. The acute phase parameters C-reactive protein (CRP) and serum amyloid A (SAA) increased in parallel with COVID-19 severity reaching significance in all patients with moderate and severe COVID-19 (**Figures 1A, B**). Expression patterns of measured chemokines and cytokines are shown as median in a heatmap (**Supplementary Figure 1**). Baseline TNF*α* in plasma samples was increased in HD patients compared to non-HD patients. The levels increased further along with COVID-19 severity, but only in non-HD patients significance was reached (**Figure 1C**). IL-27, IL-17A and IL-12 plasma levels were increased in SARS-CoV-2 negative HD patients compared to healthy controls (**Figures 1D–F**). IL-27, IL-17A and IL-12 plasma levels increased in COVID-19 non-HD patients depending on disease severity, whereas no significant dynamics were detected in HD patients (**Figures 1D–F**). Interestingly, TNF*α*, IL-27, IL-17A and IL-12 plasma levels of SARS-CoV-2 negative HD patients were in a similar range as in moderate and severe COVID-19 non-HD patients (**Figures 1C–F**).

### Decreased CD3^+^, CD4^+^ and CD8^+^ T Cells Are Hallmarks of Moderate/Severe COVID-19 in All Patients

**Figure 2:**
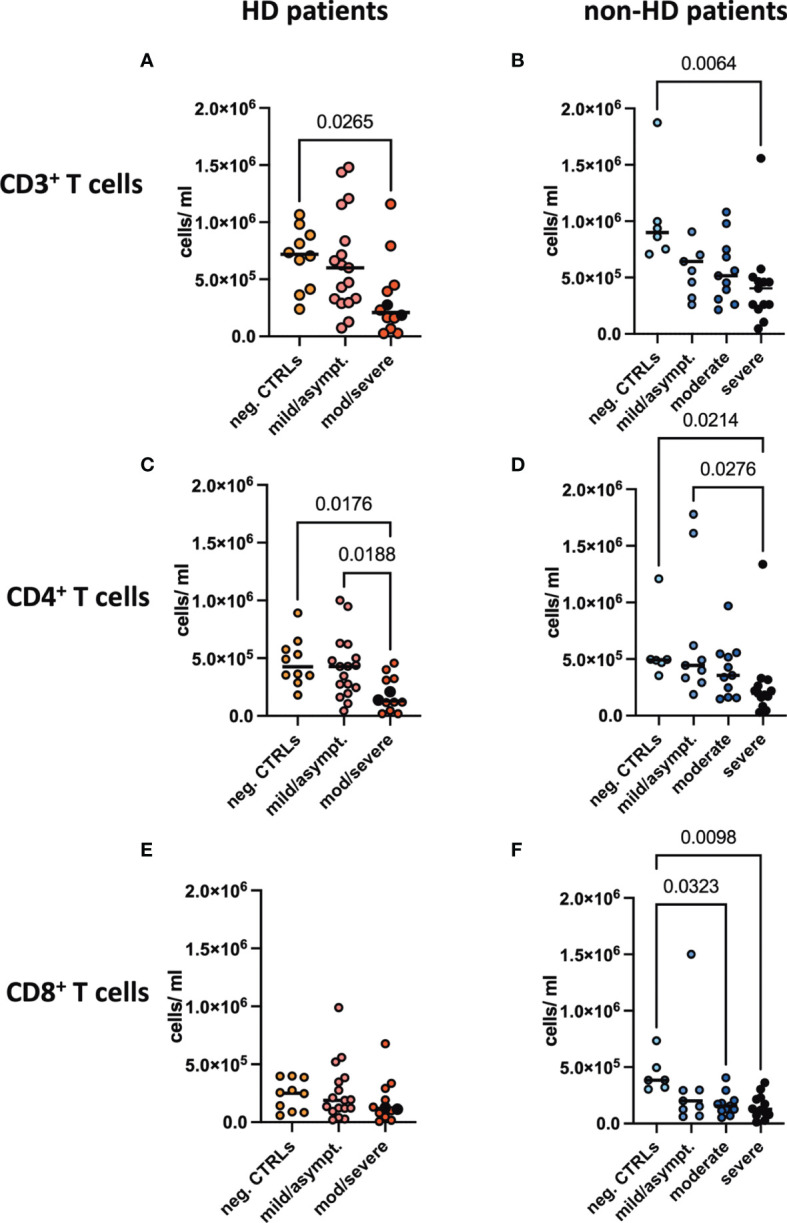
Absolute T cell numbers in peripheral blood. Absolute numbers of **(A, B)** CD3^+^, **(C, D)** CD4^+^ and **(E, F)** CD8^+^ T cells were evaluated in the peripheral blood of **(A, C, E)** HD- and **(B, D, F)** non-HD patients without SARS-CoV-2 infection (orange circles, HD: n = 10; light blue circles, non-HD: 6), with mild/asymptomatic (light red circles, HD: n = 18; mid blue circles, non-HD: n = 9), moderate (red circles, HD: n = 11; blue circles, non-HD: n = 11) and severe COVID-19 disease (black circles, HD: n = 2; dark blue circles, non-HD: n = 13). Severe and moderate COVID-19 HD patients were analyzed as one group. Data are shown as individual values as well as median.

T cell subpopulations were absolutely quantified in the six different patient groups. Since we detected substantial differences in immune cell subsets between the HD and non-HD patients without SARS-CoV-2 infection, as described previously (7), we analyzed them separately. Absolute numbers of CD3^+^ T cells were found to be decreased in moderate/severe COVID-19 HD patients and in severe COVID-19 non-HD patients (**Figures 2A, B**). CD4^+^ T cells were decreased in moderate/severe COVID-19 HD patients and in severe COVID-19 non-HD patients (**Figures 2C, D**). CD8^+^ T cells showed a trend for decreased numbers in the moderate/severe COVID-19 HD patients (**Figure 2E**), whereas this population was significantly decreased in the moderate and severe non-HD COVID-19 population (**Figure 2F**). Of note, relative numbers of respective T cell populations in lymphocytes were not significantly regulated except for decreased frequencies of CD3^+^ T cells in moderate/severe COVID-19 HD patients (data not shown).

### Regulatory T Cells Decreased in Peripheral Blood of Moderate/Severe COVID-19 Patients

**Figure 3:**
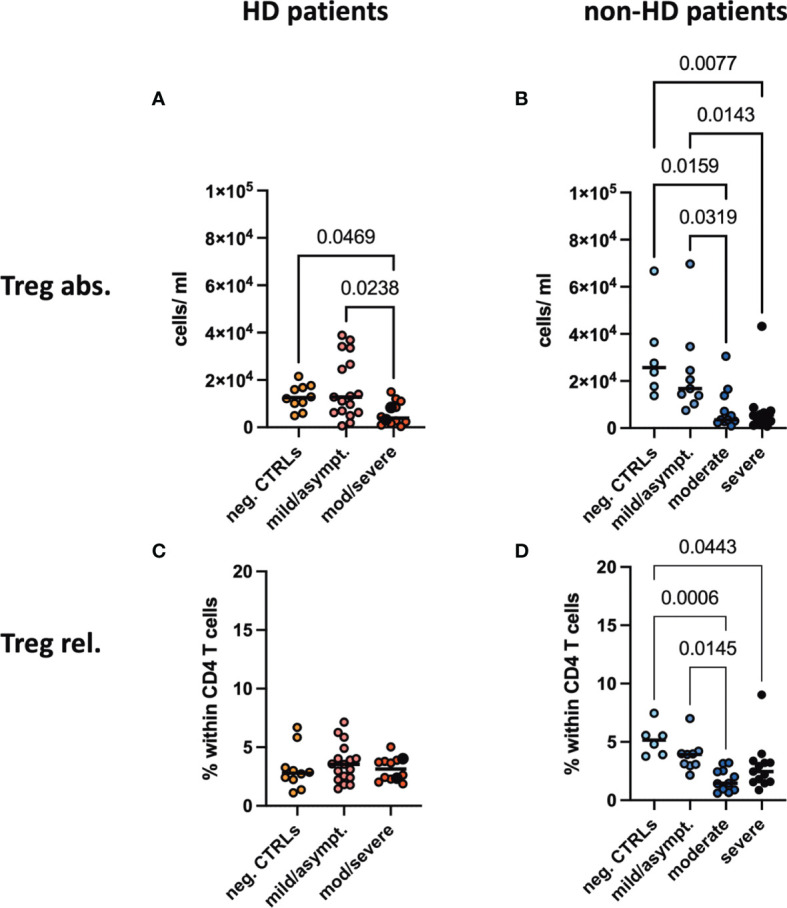
*Absolute numbers and frequencies of Tregs in peripheral blood.* **(A, B)** Absolute numbers and **(C, D)** frequencies of regulatory T cells (Tregs) were evaluated in the peripheral blood of **(A, C)** HD- and **(B, D)** non-HD patients without SARS-CoV-2 infection (orange circles, HD: n = 10; light blue circles, non-HD: 6), with mild/asymptomatic (light red circles, HD: n = 18; mid blue circles, non-HD: n = 9), moderate (red circles, HD: n = 11; blue circles, non-HD: n = 11) and severe COVID-19 disease (black circles, HD: n = 2; dark blue circles, non-HD: n = 13). Severe and moderate COVID-19 HD patients were analyzed as one group. Data are shown as individual values as well as median.

Regulatory T cells characterized by CD4^+^CD25^hi^FoxP3^+^ were decreased in absolute and relative numbers in the SARS-CoV-2 negative HD population compared to healthy controls. In patients with moderate/severe COVID-19, absolute Treg numbers decreased significantly both in the HD and non-HD cohorts (**Figures 3A, B**). Frequencies of CD25^hi^FoxP3^+^ in CD4^+^ T cells were significantly decreased in the moderate and severe COVID-19 non-HD population, but not in the COVID-19 HD population (**Figures 3C, D**).

### CD38^+^CD8^+^ Effector Memory and TEMRA T Cells as Well as CD161^+^CD8^+^ T Cells Increased Only in COVID-19 HD Patients

**Figure 4:**
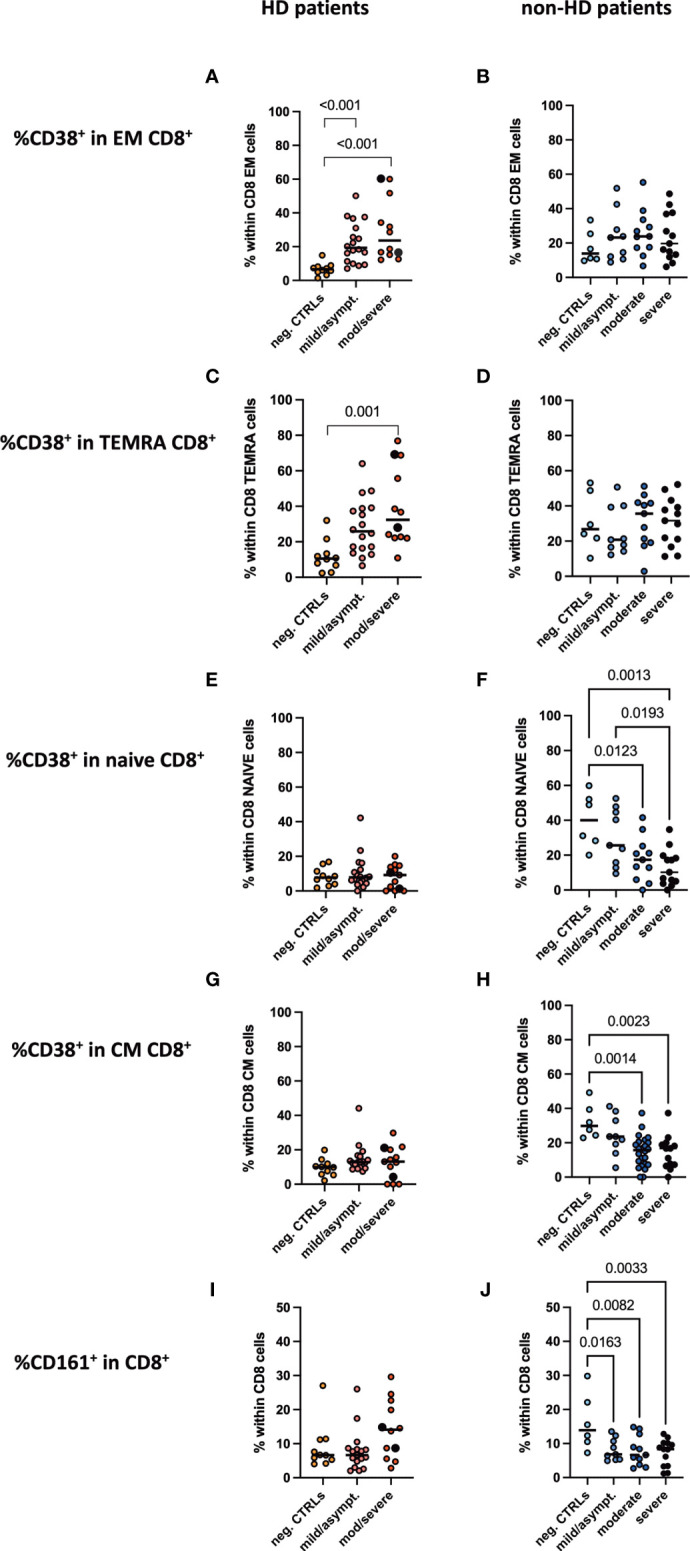
CD38^+^CD8^+^ activated T cell populations and CD161^+^CD8^+^ T cells in peripheral blood. Frequencies of CD38^+^ positive cells in **(A, B)** CD8^+^ effector memory, **(C, D)** CD8^+^ TEMRA, **(E, F)** CD8^+^ naïve, **(G, H)** CD8^+^ central memory T cells as well as frequencies of **(I, J)** CD161^+^CD8^+^ T cells were measured in the peripheral blood of **(A, C, E, G, I)** HD- and **(B, D, F, H, J)** non-HD patients without SARS-CoV-2 infection (orange circles, HD: n = 10; light blue circles, non-HD: 6), with mild/asymptomatic (light red circles, HD: n = 18; mid blue circles, non-HD: n = 9), moderate (red circles, HD: n = 11; blue circles, non-HD: n = 11) and severe COVID-19 disease (black circles, HD: n = 2; dark blue circles, non-HD: n = 13). Severe and moderate COVID-19 HD patients were analyzed as one group. Data are shown as individual values as well as median.

Frequencies of CD4^+^ and CD8^+^ subpopulations, namely naïve, effector memory, central memory and TEMRA CD4^+^ CD8^+^ T cells, were evaluated. We only detected a decrease in effector memory CD4^+^ and CD8^+^ T cells in moderate/severe COVID-19 HD patients, while all other subpopulations and groups displayed no significant difference (**Supplementary Figures 2, 3**). We studied various markers for CD4 and CD8 T cells as outlined in our methods in an unbiased approach. The activation marker CD38 was found to be significantly regulated between the groups. Interestingly, we detected a significant increase in the CD38^+^CD8^+^ effector memory and TEMRA T cell population in the moderate/severe COVID-19 HD cohort, which was not the case in the COVID-19 non-HD patients (**Figures 4A–D**). Frequencies of CD38^+^ cells in CD8^+^ naïve and central memory CD8^+^ T cells decreased significantly in moderate and severe COVID-19 non-HD patients, but not in moderate/severe COVID-19 HD patients (**Figures 4E–H**). Furthermore, frequencies of CD161^+^ cells in CD8^+^ T cells were significantly decreased in COVID-19 positive non-HD patients independent on severity of disease, which was not seen in COVID-19 positive HD patients (**Figures 4I, J**).

### B Cell Subpopulations Showed Comparable Dynamics in All COVID-19 Patients

**Figure 5:**
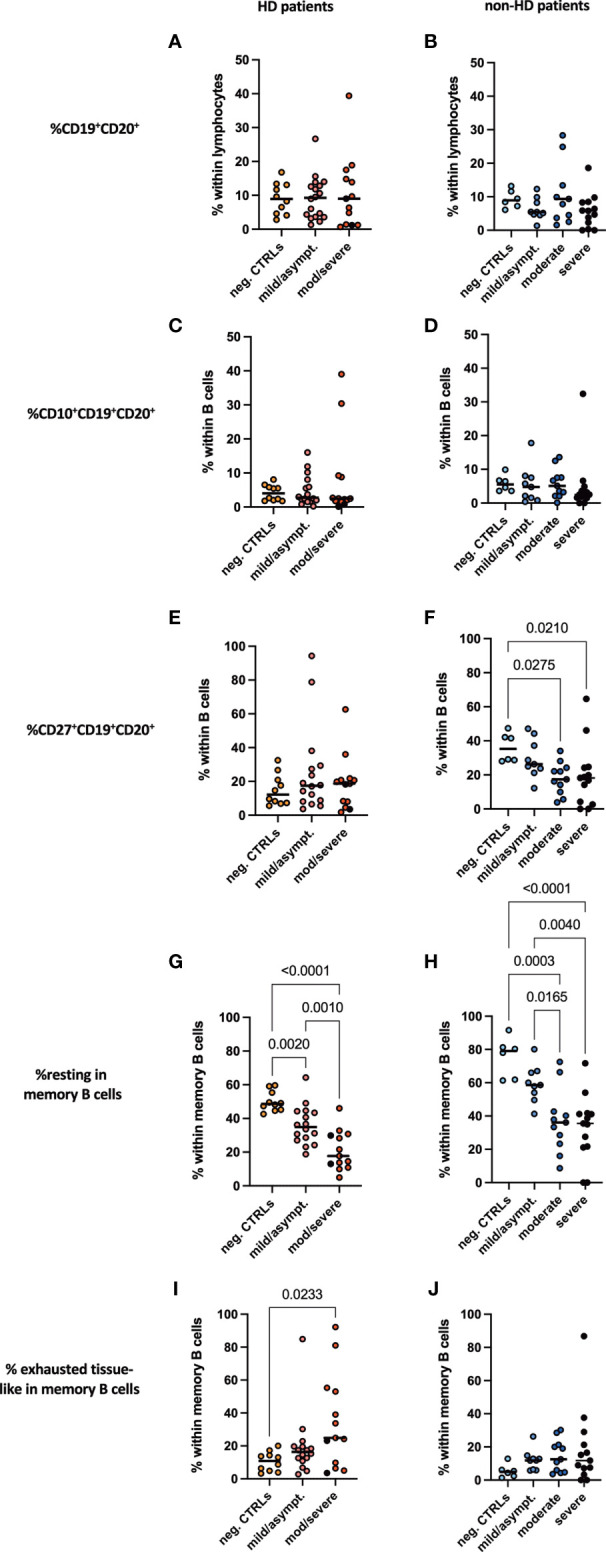
B cell subpopulations in peripheral blood. Frequencies of **(A, B)** CD19^+^CD20^+^ B cells, **(C, D)** CD10^+^CD19^+^CD20^+^, **(E, F)** CD27^+^CD19^+^CD20^+^, **(G, H)** resting memory B cells and **(I, J)** exhausted tissue-like memory B cells measured in peripheral blood of **(A, C, E, G, I)** HD- and **(B, D, F, H, J)** non-HD patients without SARS-CoV-2 infection (orange circles, HD: n = 10; light blue circles, non-HD: 6), with mild/asymptomatic (light red circles, HD: n = 18; mid blue circles, non-HD: n = 9), moderate (red circles, HD: n = 11; blue circles, non-HD: n = 11) and severe COVID-19 disease (black circles, HD: n = 2; dark blue circles, non-HD: n = 13). Severe and moderate COVID-19 HD patients were analyzed as one group. Data are shown as individual values as well as median.

No difference was seen in frequencies of CD19^+^CD20^+^ pan B cells in lymphocytes neither between HD and non-HD patients nor in patients with or without SARS-CoV-2 infection (**Figures 5A, B**). This held true for early developmental CD10^+^ B cells (**Figures 5C, D**). Moderate and severe COVID-19 resulted in a significant decrease in CD27^+^ B cells, which account for memory B cells, in non-HD patients, which was not the case for HD patients with COVID-19 (**Figures 5E, F**). Percentages of unswitched (CD19^+^CD20^+^CD10^-^IgD^+^CD38^-^), transitional (CD19^+^CD20^+^CD10^+^IgD^+^CD38^+^), memory (CD19^+^CD20^+^CD10^-^IgD^-^CD38^-^CD27^+^), class switched memory (CD19^+^CD20^+^CD27^+^IgM^-^IgD^-^) and IgD post switched B cells (CD19^+^CD20^+^IgD^-^CD27^+^) did not differ between the groups (**Supplementary Figure 4**). Again, all memory B cell populations were found to be decreased in the HD population without SARS-CoV-2 infection as compared to HD patients with COVID-19 (**Supplementary Figure 4**). Interestingly, resting memory B cells, characterized by frequencies of CD19^+^CD20^+^CD10^-^IgD^-^CD38^-^CD21^+^CD27^+^ cells were significantly decreased in moderate and severe COVID-19 both HD and non-HD patients (**Figures 5G, H**). In contrast, exhausted tissue like memory B cells increased significantly in HD patients with moderate/severe COVID-19 (**Figure 5I**). These cells tended to increase in moderate and severe COVID-19 non-HD patients without reaching significance (**Figure 5J**).

## Discussion

SARS-CoV-2 infection is associated with high mortality rates, especially in patients with comorbidities such as obesity, diabetes mellitus type 2, hypertension and CKD (1). Thus, patients treated with renal replacement therapy such as HD, who frequently suffer from all these diseases, are thought to have increased mortality from COVID-19. Nevertheless, recent reports suggest that disease severity, mortality as well as ICU admissions might not be as high as expected in patients with need for HD (2, 6). In the study presented, we add to this evidence that HD patients do not show excess mortality and further describe their immune cell composition in peripheral blood as well as cytokine and chemokine plasma profile. Thereby, we provide evidence that HD patients show chronic inflammation at baseline and that this inflammation does not significantly increase in case of COVID-19 independent of its severity. This is in sharp contrast to non-HD patients, who develop significant increases in Th1 and Th17 cytokine plasma levels dependent on COVID-19 severity. Remarkably, HD-patients without SARS-CoV-2 infection show cytokine levels as high as non-HD patients with moderate and severe COVID-19. Interestingly, this is not applicable to acute phase parameters such as CRP and serum amyloid A plasma levels, which both show a similar profile in HD and non-HD patients and clearly increase with disease severity. Low-grade inflammation in HD patients and especially high cytokine levels in HD patients are well described (16–18) and have been linked to high mortality and development of atherosclerosis in this patient cohort (17, 19–22). It might be speculated that this chronic inflammation, which mainly affects innate immunity, might improve initial innate immune response and outcome of HD patients with COVID-19. Recently, it has been proposed that innate immunity is pivotal to prevent severe COVID-19 by limiting viral spread and activating adaptive immunity as early as possible (9). When characterizing adaptive immunity, we found prominent decreases of pan T cells, CD4^+^ and CD8^+^ T cells in patients with moderate/severe COVID-19 independent from the necessity of HD treatment. Decreased T cell counts are a well-known hallmark of severe COVID-19 (23, 24). Interestingly, CD4^+^ and CD8^+^ subpopulations remained largely unaltered in their frequencies. Only effector memory CD4^+^ and CD8^+^ were decreased in their frequencies within the CD4^+^ and CD8^+^ T cells in patients with moderate/severe COVID-19, whereas naïve, central memory and TEMRA subpopulations remained unchanged. Of note, we saw an impressive reduction especially in CD4^+^CD25^+^FoxP3^+^ regulatory T cells (Tregs) in moderate/severe COVID-19 patients. The role of Tregs in COVID-19 is still under investigation with some reports describing increased (25, 26) or decreased Treg numbers or frequencies (27–30). This might be largely attributable to the markers used to describe Tregs, but also to the severity of disease and time point of evaluation (31). Noteworthy, two critically ill patients treated with cord-blood derived Tregs seemed to improve significantly after Treg transfer (32).

By performing an unbiased analysis of respective markers and populations, we detected a significant increase in CD38^+^CD8^+^ effector memory and TEMRA T cells only in HD patients depending on their COVID-19 severity. CD38 is an activation marker of T cells. CD38^+^CD8^+^ T cells play a crucial role in virus control and elimination since they exhibit high effector function such as cytotoxicity (33). They have also been implicated to be more prone to cell death and are key for establishing a robust memory response (34). Our data are in accordance with others showing increased levels of CD38^+^CD8^+^ T cells in the viral clearance phase of COVID-19 patients (24, 35). Interestingly, we saw a different CD38 regulation dependent on CD8^+^ T cell subpopulations. CD8^+^ effector memory T cells are highly cytotoxic and mainly present in the circulation thereby being easily recruited to sites of inflammation (36). In parallel, TEMRA CD8^+^T cells are a preformed effector population, which can be easily activated upon T cell or cytokine stimulation (37). Both of them showed increased CD38^+^ expression in HD patients already with mild/asymptomatic COVID-19 and even more with moderate/severe COVID-19, thus providing an effective response to the viral infection and thereby being one explanation for the improved outcome in this cohort. Furthermore, CD161^+^ expressing CD8^+^ T cells were significantly decreased in peripheral blood in moderately/severely diseased non-HD patients, which is in accordance with a previous publication (38). Most interestingly, HD patients did not show this prominent decrease, but rather showed a slight increase in CD161^+^CD8^+^ T cells in moderate/severe COVID-19 patients. CD161^high^ expressing CD8^+^ T cells produce high amounts of IL-17 and are called Tc17 cells, whereas CD161^int^ expressing CD8^+^ T cells do not secrete IL-17 but are reported to be a unique population of memory CD8^+^ cells with enhanced effector functions (39). Together, both populations are key to limit viral infections such as COVID-19 and might thus contribute to the improved phenotype in HD patients.

A robust CD4 T cell response is needed to activate B cells that transform into plasma cells and plasmablast, which produce specific antibodies to fight viral infection (23). Increased plasmablast numbers were found patients with severe COVID-19 especially in the inflammatory phase of disease shown by single cell RNAseq technology (40). HD patients are well known to have an altered B cell immunity (41, 42), which might be associated with mortality (42). In our hands, B cells characterized by CD19 and CD20 positivity did not change in frequencies neither between non-HD and HD patients nor between disease severity. Interestingly, CD27^+^ expressing B cells, which are known to have memory functions (43), decreased significantly in non-HD patients depending on disease severity, which was not seen in HD-patients. Resting memory B cells decreased in moderate/severe COVID-19 patients, whereas exhausted tissue-like memory B cells increased. A decrease of resting memory B cells has also been shown for other viral infections such as HIV (44). Exhausted tissue like memory B cells increase in HIV infected patients and severe COVID-19 patients and contribute to a diminished antibody response in infected patients (45, 46). Both populations are key to fight COVID-19 and were altered in moderate/severe COVID-19 patients independently from the presence/absence of hemodialysis treatment. Thus, B cells do not differ largely between HD and non-HD patients and do not seem to explain the differences in COVID-19 outcomes.

Our study has several limitations, which need to be taken into account when interpreting the data. Firstly, we do not have serial evaluations of the patients during the course of disease. Thus, differences in measured parameters might arise from dynamics of immune cells in peripheral blood during COVID-19. A further study focusing on the course of disease from the time-point of infection or hospitalization in non-HD and HD patients is needed. Secondly, the patient number is not powered for hard endpoints such as mortality or ICU admission. Therefore, clinical outcomes in our cohort need to be carefully interpreted. Thirdly, we did not have the possibility to track SARS-CoV-2 specific T and B cell responses in our patients. Fourthly, we recruited more non-HD patients with severe COVID-19 phenotype compared to the HD population. Thus, our data give limited information about the cytokine and immune cell status of HD patients with severe COVID-19. The strength of our study is the sizable number of COVID-19 patients with asymptomatic/mild disease, while most of the studies so far recruited mainly hospitalized COVID-19 patients. Furthermore, this is to our knowledge the first study evaluating immune mechanisms in HD patients with SARS-CoV-2 infection.

Together, we see an improved outcome in our HD cohort with less ICU admissions, which might be attributed to a chronic inflammatory state and increased frequencies of CD38^+^CD8^+^ memory and CD161^+^CD8^+^ T cells, both having a high cytotoxic activity to limit COVID-19 severity.

## Data Availability Statement

The original contributions presented in the study are included in the article/**Supplementary Material**. Further inquiries can be directed to the corresponding author.

## Ethics Statement

The studies involving human participants were reviewed and approved by the ethical board of the Medical University of Graz (32-323 ex19/20). The patients/participants provided their written informed consent to participate in this study.

## Author Contributions

BP: conception of study, data collection, data analysis, interpretation, drafting article, and final approval of manuscript. BO: data collection, data analysis and interpretation, drafting the article, and final approval of manuscript. AK: data collection, revision of the article, and final approval of manuscript. KA: data collection, revision of the article, and final approval of manuscript. ME: data collection, revision of the article, and final approval of manuscript. SSc: data collection, revision of the article, and final approval of manuscript. VP: data collection, data analysis, drafting the article, and final approval of manuscript. SSt: data analysis, revision of the article, and final approval of manuscript. AE: data analysis, revision of the article, and final approval of manuscript. RR: data analysis, revision of the article, and final approval of manuscript. TP: data interpretation, revision of the article, and final approval of manuscript. AR: data interpretation, revision of the article, and final approval of manuscript. MB: data collection, critical revision of the article, and final approval of manuscript. PE: conception of study, data collection, interpretation, revision of the article, and final approval of manuscript. KE: conception of study, data collection, data analysis, interpretation, drafting the article, and final approval of manuscript. All authors contributed to the article and approved the submitted version.

## Funding

This work was supported by the Austrian Science Funds (FWF) to KE (MOLIN PhD program W1241) and the Austrian National Bank OeNB (Nr.17212 to KE) as well as by an investigator-initiated research grant by Chiesi to KE. Work done in the Center for Biomarker Research in Medicine was funded by the Austrian Federal Government within the COMET K1 Centre Program, Land Steiermark and Land Wien.

## Conflict of Interest

AE and RR were employed by Joanneum Research Forschungsgesellschaft mbH.

The remaining authors declare that the research was conducted in the absence of any commercial or financial relationships that could be construed as a potential conflict of interest.

## Publisher’s Note

All claims expressed in this article are solely those of the authors and do not necessarily represent those of their affiliated organizations, or those of the publisher, the editors and the reviewers. Any product that may be evaluated in this article, or claim that may be made by its manufacturer, is not guaranteed or endorsed by the publisher.
