# Supplementary material for: Chronic Inflammation Might Protect Hemodialysis Patients From Severe COVID-19
Source: Front Immunol. 2022 Feb 21;13:821818. doi: 10.3389/fimmu.2022.821818 (PMC8901184; doi:10.3389/fimmu.2022.821818)

SUPPLEMENTAL FIGURES

Supplemental figure 1. *Heat map of measured cytokines*. Cytokine plasma levels were measured HD- and non-HD patients without SARS-CoV-2 infection (HD: n=6; non-HD: 6), with mild/asymptomatic (HD: n=18; non-HD: n=9), moderate (non-HD: n=11) and severe COVID-19 disease (non-HD: n=13). Severe and moderate COVID-19 HD patients (n=13) were analyzed as one group. Data are shown as median of respective groups.

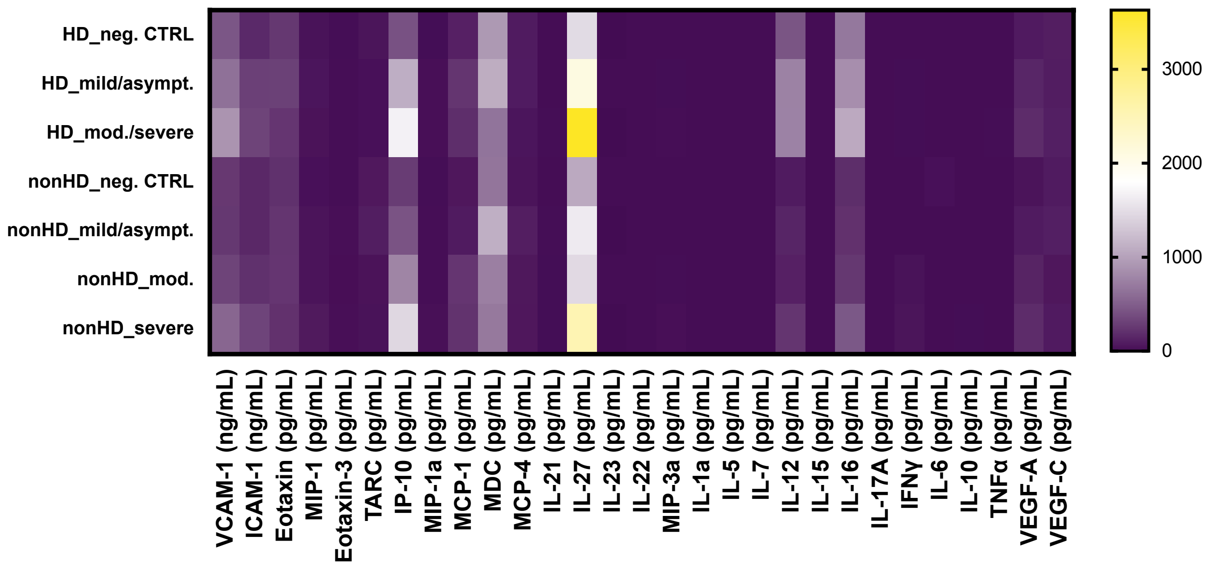

Supplemental figure 2. *CD4$^+$ T cell subpopulations in peripheral blood.* Frequencies of (A,B) CD4$^+$ naïve, (C,D) CD4$^+$ effector memory, (E,F) CD4$^+$ central memory, (G,H) CD4$^+$ TEMRA T cells were measured in the peripheral blood of (A,C,E,G) HD- and (B,D,F,H) non-HD patients without SARS-CoV-2 infection (HD: n=10; non-HD: 6), with mild/asymptomatic (HD: n=18; non-HD: n=9), moderate (red circles, HD: n=11; blue circles, non-HD: n=11) and severe COVID-19 (black circles, HD: n=2; dark blue circles, non-HD: n=13) were included. Severe and moderate COVID-19 HD patients were analyzed as one group. Data are shown as individual values as well as median.

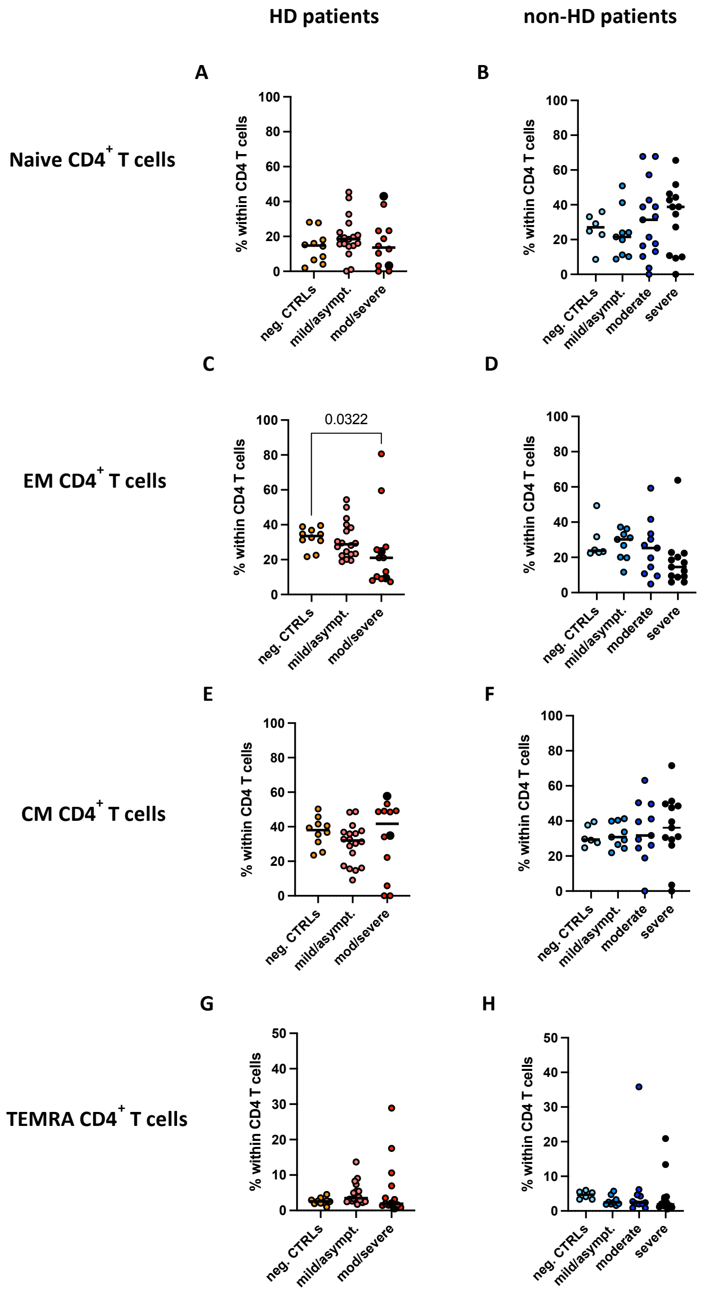

Supplemental figure 3. *CD8⁺ T cell subpopulations in peripheral blood.* Frequencies of (A,B) CD8⁺ naïve, (C,D) CD8⁺ effector memory, (E,F) CD8⁺ central memory, (G,H) CD8⁺ TEMRA T cells were measured in the peripheral blood of (A,C,E,G) HD- and (B,D,F,H) non-HD patients without SARS-CoV-2 infection (HD: n=10; non-HD: 6), with mild/asymptomatic (HD: n=18; non-HD: n=9), moderate (red circles, HD: n=11; blue circles, non-HD: n=11) and severe COVID-19 (black circles, HD: n=2; dark blue circles, non-HD: n=13) were included. Severe and moderate COVID-19 HD patients were analyzed as one group. Data are shown as individual values as well as median.

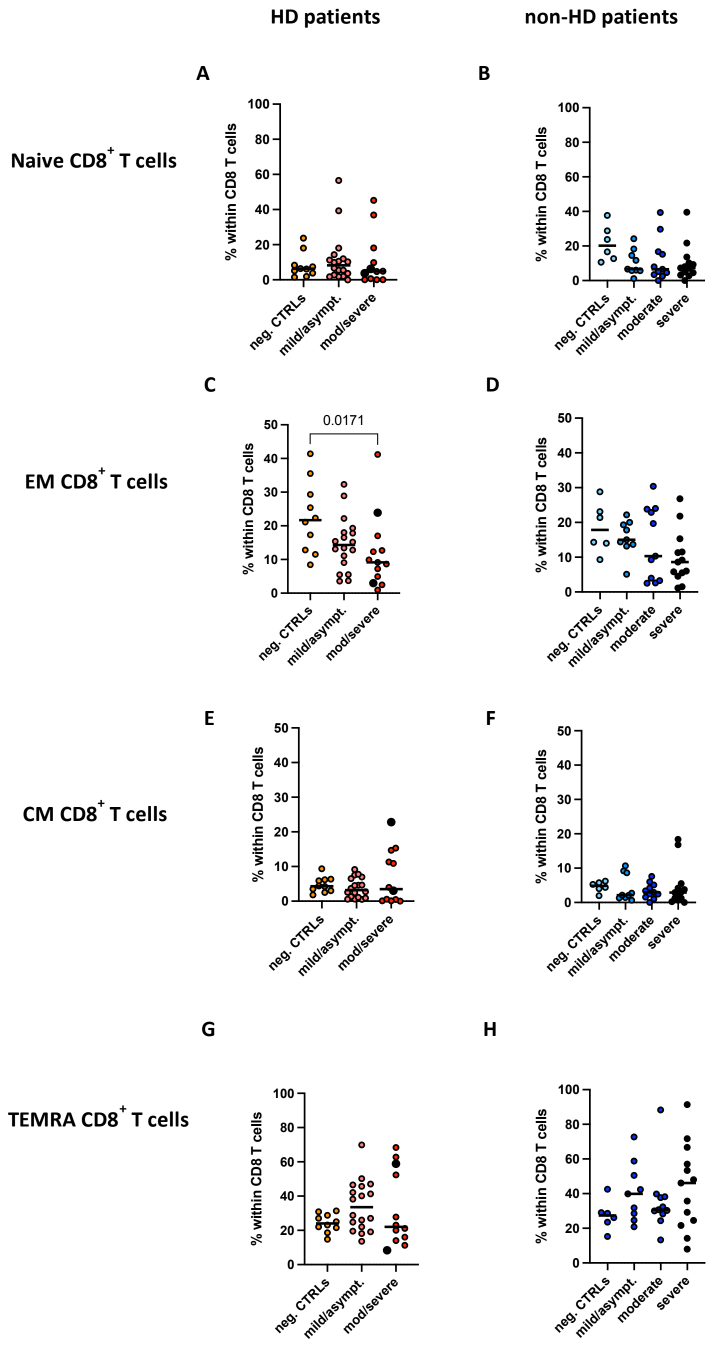

Supplemental figure 4. *B cell subpopulations in peripheral blood.* Frequencies of (A,B) unswitched, (C,D) transitional, (E,F) memory, (G,H) class-switched memory, (I,J) IgD-post switched memory B cells were measured in the peripheral blood of (A,C,E,G) HD- and (B,D,F,H) non-HD patients without SARS-CoV-2 infection (HD: n=10; non-HD: 6), with mild/asymptomatic (HD: n=18; non-HD: n=9), moderate (red circles, HD: n=11; blue circles, non-HD: n=11) and severe COVID-19 (black circles, HD: n=2; dark blue circles, non-HD: n=13) were included. Severe and moderate COVID-19 HD patients were analyzed as one group. Data are shown as individual values as well as median.

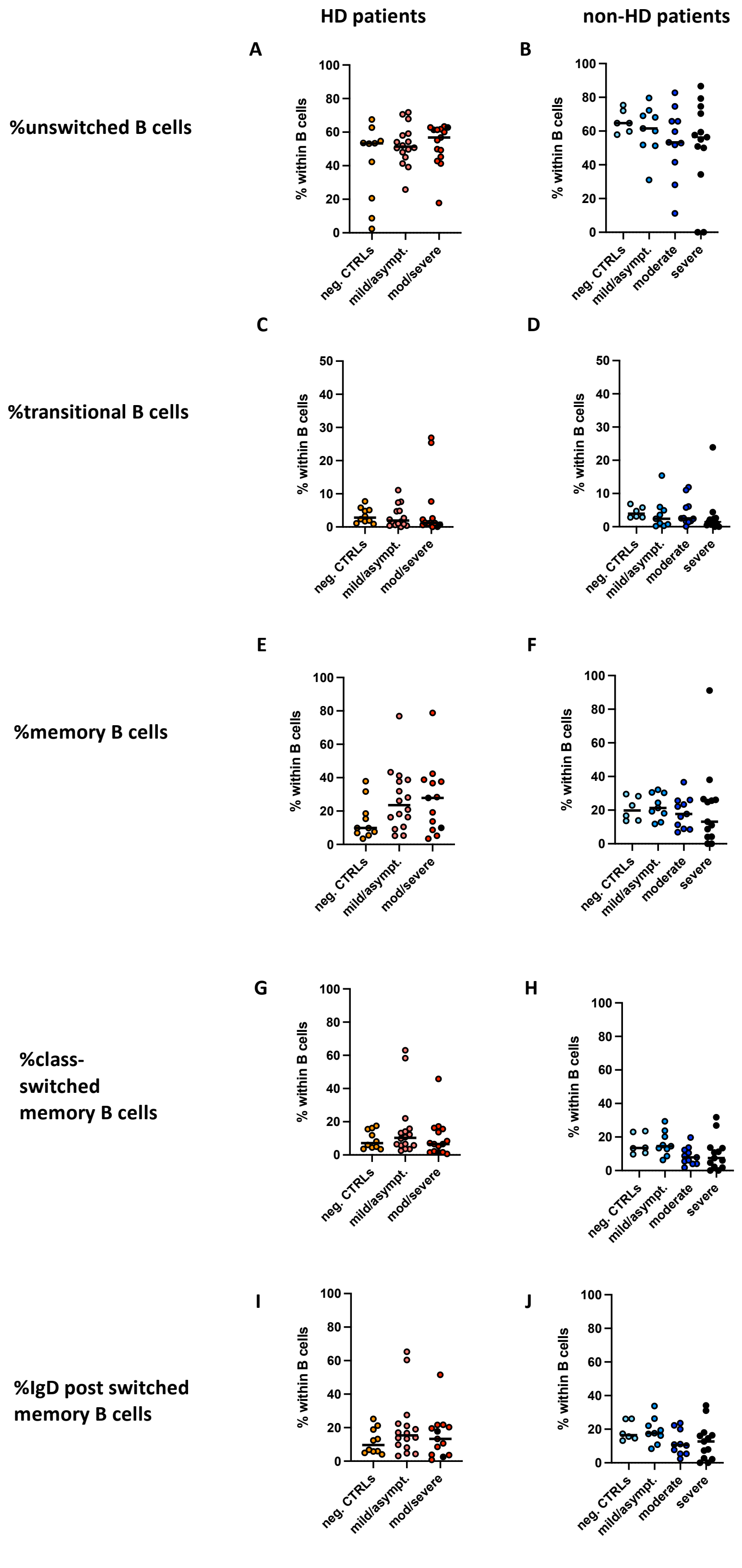

Supplement: Supplementary file 1 [file DataSheet_1.pdf]
