# Supplementary material for: Chronic Inflammation Might Protect Hemodialysis Patients From Severe COVID-19
Source: Front Immunol. 2022 Feb 21;13:821818. doi: 10.3389/fimmu.2022.821818 (PMC8901184; doi:10.3389/fimmu.2022.821818)
Supplement: Supplementary file 2 [file Table_1.pdf]

Supplemental table 1. FACS antibody details.

Antibody	Clone	Fluorochrome	Manufacturer	ref#
CD3	SK7	APC-H7	Becton Dickinson	560176
CD4	RPA-T4	BV605	Becton Dickinson	562659
CD45RA	HI100	BV786	Becton Dickinson	563870
CD15S	CSLEX1	BV421	Becton Dickinson	563912
Ki-67	B56	AF488	Becton Dickinson	558616
FoxP3	259D/C7	PE	Becton Dickinson	560046
CD8	RPA-T8	BV711	Becton Dickinson	563677
CD161	DX12	APC	Becton Dickinson	550968
CD127	HIL-7R-M21	BV510	Becton Dickinson	563086
Fixable Viability Dye		APC-R700	Becton Dickinson	564997
CD25	2A3	PE-Cy7	Becton Dickinson	335824
CD39	TU66	PE-CF594	Becton Dickinson	563678
CD147	HIM6	PerCP-Cy5.5	Becton Dickinson	562554
CD3	SP34-2	AF700	Becton Dickinson	561805
CD4	RPA-T4	PE-CF594	Becton Dickinson	562316
CD27	M-T271	APC-H7	Becton Dickinson	560222
CD57	HNK-1	FITC	Becton Dickinson	333169
PD-1 (CD279)	EH12.1	PE	Becton Dickinson	560795
CCR7 (CD197)	3D12	AF647	Becton Dickinson	557734
HLA-DR	G46-6	BV650	Becton Dickinson	564231
CD127	HIL-7R-M21	PerCpCy5.5	Becton Dickinson	560551
CD95	DX2	SuperBright 600	ThermoFisher	63-0959-41
CD38	HIT2	BV421	Becton Dickinson	562445
CD28	CD28.2	BV510	Becton Dickinson	563075
CD19	SJ25C1	PE	Becton Dickinson	345789
CD20	L27	APC-H7	Becton Dickinson	641414
IgD	IA6-2	PerCp-Cy5.5	Becton Dickinson	561315
IgM	G20-127	BB515	Becton Dickinson	564622
CD24	ML5	BV711	Becton Dickinson	563401
CD27	L128	BV786	Becton Dickinson	563327
CD86	2331 (FUN-1)	PE-CF594	Becton Dickinson	562390
CD5	L17F12	PE-Cy7	Becton Dickinson	348810
CD38	HIT2	APC-R700	Becton Dickinson	564979
CD10	HI10a	BV510	Becton Dickinson	563032
CD21	B-ly4	BV421	Becton Dickinson	566260
