# Supplementary material for: Chronic Inflammation Might Protect Hemodialysis Patients From Severe COVID-19
Source: Front Immunol. 2022 Feb 21;13:821818. doi: 10.3389/fimmu.2022.821818 (PMC8901184; doi:10.3389/fimmu.2022.821818)
Supplement: Supplementary file 3 [file Table_2.pdf]

Supplemental table 2. FACS gating strategy.

| **Panel 1** | | | |
|---|---|---|---|
| **Population** | **reported as % of** | **Phenotype** | **Gating** |
| %CD3+ T cells | lymphocytes | CD3+ | CD3 histogram |
| %CD4+ T cells | lymphocytes | CD4+ | CD4 histogram/SSC-A |
| %CD8+ T cells | lymphocytes | CD8+ | CD8 histogram/SSC-A |
| %CD3+CD4+ T cells | lymphocytes | CD3+CD4+ | CD4 vs. CD3 |
| %CD3+CD8+ T cells | lymphocytes | CD3+CD8+ | CD8 vs. CD3 |
| %CD8-CD4- cells | CD3 T cells | CD3+CD4-CD8- | CD4 vs. CD8 |
| %CD8-CD4+ cells | CD3 T cells | CD3+CD4+CD8- | CD4 vs. CD8 |
| %CD8+CD4- cells | CD3 T cells | CD3+CD4-CD8+ | CD4 vs. CD8 |
| %CD8+CD4+ cells | CD3 T cells | CD3+CD4+CD8+ | CD4 vs. CD8 |
| %CD25+FoxP3+ Tregs | CD3+CD4+ T cells | CD3+CD4+CD25+Foxp3+ | FoxP3 vs. CD25 |
| %CD25+FoxP3- | CD3+CD4+ T cells | CD3+CD4+CD25+Foxp3- | FoxP3 vs. CD25 |
| %Treg effector (CD45RA-CD15s+) | Tregs | CD3+CD4+CD25+Foxp3+CD45RA-CD15s+ | CD45RA vs. CD15s |
| %Treg naive (CD45RA+CD15s-) | Tregs | CD3+CD4+CD25+Foxp3+CD45RA+CD15s- | CD45RA vs. CD15s |
| %CD45RA-CD15s- | Tregs | CD3+CD4+CD25+Foxp3+CD45RA-CD15s- | CD45RA vs. CD15s |
| %CD45RA+CD15s+ | Tregs | CD3+CD4+CD25+Foxp3+CD45RA+CD15s+ | CD45RA vs. CD15s |
| %Treg effector proliferative (CD45RA-KI67+) | Tregs | CD3+CD4+CD25+Foxp3+CD45RA-Ki67+ | CD45RA vs. Ki67 |
| %CD45RA-KI67- | Tregs | CD3+CD4+CD25+Foxp3+CD45RA-Ki67- | CD45RA vs. Ki67 |
| %CD45RA+KI67+ | Tregs | CD3+CD4+CD25+Foxp3+CD45RA+Ki67+ | CD45RA vs. Ki67 |
| %CD45RA+KI67- | Tregs | CD3+CD4+CD25+Foxp3+CD45RA+Ki67- | CD45RA vs. Ki67 |

| | | | |
|---|---|---|---|
| %CD147high Tregs (CD45RA-CD147++ high) | Tregs | CD3+CD4+CD25+Foxp3+CD45RA-CD147++ | CD45RA vs. CD147 |
| %CD147med Tregs (CD45RA-CD147+ medium) | Tregs | CD3+CD4+CD25+Foxp3+CD45RA-CD147+ | CD45RA vs. CD147 |
| %CD45RA+CD147+ | Tregs | CD3+CD4+CD25+Foxp3+CD45RA+CD147+ | CD45RA vs. CD147 |
| %Distribution of CD39 on effector Treg cells (CD45RA-CD39+) | Tregs | CD3+CD4+CD25+Foxp3+CD45RA-CD39+ | CD45RA vs. CD39 |
| %CD45RA-CD39- | Tregs | CD3+CD4+CD25+Foxp3+CD45RA-CD39- | CD45RA vs. CD39 |
| %CD45RA+CD39+ | Tregs | CD3+CD4+CD25+Foxp3+CD45RA+CD39+ | CD45RA vs. CD39 |
| %CD45RA+CD39- | Tregs | CD3+CD4+CD25+Foxp3+CD45RA+CD39- | CD45RA vs. CD39 |
| Expression % of CD147++high on effector Tregs | CD45RA-CD15s+Tregs | CD3+CD4+CD25+Foxp3+CD45RA-CD15s+CD147++ | CD147 vs. SSC |
| Expression % of CD147+med on effector Tregs | CD45RA-CD15s+Tregs | CD3+CD4+CD25+Foxp3+CD45RA-CD15s+CD147+ | CD147 vs. SSC |
| Expression % of CD39 on effector Treg cells | CD45RA-CD15s+Tregs | CD3+CD4+CD25+Foxp3+CD45RA-CD15s+CD39+ | CD39 vs. SSC |
| Expression % of Ki67 on effector Treg cells | CD45RA-CD15s+Tregs | CD3+CD4+CD25+Foxp3+CD45RA-CD15s+Ki67+ | KI67 vs. SSC |
| %CD161- trans-Tregs | CD45RA-CD15s- | CD3+CD4+CD25+Foxp3+CD161- | CD161 vs. SSC-A |
| %CD161+ Th17-type Tregs | CD45RA-CD15s- | CD3+CD4+CD25+Foxp3+CD161+ | CD161 vs. SSC-A |
| Th cell Proliferation ( %CD45RA-KI67+) | CD3+CD4+ T cells | CD3+CD4+CD45RA-Ki67+ | CD45RA vs. Ki67 |
| %CD45RA-KI67- | CD3+CD4+ T cells | CD3+CD4+CD45RA-Ki67- | CD45RA vs. Ki67 |
| %CD45RA+KI67+ | CD3+CD4+ T cells | CD3+CD4+CD45RA+Ki67+ | CD45RA vs. Ki67 |

| | | | |
|---|---|---|---|
| %CD45RA+KI67- | CD3+CD4+ T cells | CD3+CD4+CD45RA+Ki67- | CD45RA vs. Ki67 |
| Distribution of CD147 on Th cells ( %CD45RA-CD147++high) | CD3+CD4+ T cells | CD3+CD4+CD45RA-CD147++ | CD45RA vs. CD147 |
| %CD45RA-CD147+medium | CD3+CD4+ T cells | CD3+CD4+CD45RA-CD147+ | CD45RA vs. CD147 |
| %CD45RA+CD147+ | CD3+CD4+ T cells | CD3+CD4+CD45RA+CD147+ | CD45RA vs. CD147 |
| Distribution of CD39 on Th cells ( %CD45RA-CD39+) | CD3+CD4+ T cells | CD3+CD4+CD45RA-CD39+ | CD45RA vs. CD39 |
| %CD45RA-CD39- | CD3+CD4+ T cells | CD3+CD4+CD45RA-CD39- | CD45RA vs. CD39 |
| %CD45RA+CD39+ | CD3+CD4+ T cells | CD3+CD4+CD45RA+CD39+ | CD45RA vs. CD39 |
| %CD45RA+CD39- | CD3+CD4+ T cells | CD3+CD4+CD45RA+CD39- | CD45RA vs. CD39 |
| Tc cell Proliferation ( %CD45RA-KI67+) | CD3+CD8+ T cells | CD3+CD8+CD45RA-Ki67+ | CD45RA vs. Ki67 |
| %CD45RA-KI67- | CD3+CD8+ T cells | CD3+CD8+CD45RA-Ki67- | CD45RA vs. Ki67 |
| %CD45RA+KI67+ | CD3+CD8+ T cells | CD3+CD8+CD45RA+Ki67+ | CD45RA vs. Ki67 |
| %CD45RA+KI67- | CD3+CD8+ T cells | CD3+CD8+CD45RA+Ki67- | CD45RA vs. Ki67 |
| Distribution of CD147 on Tc cells ( %CD45RA-CD147++high) | CD3+CD8+ T cells | CD3+CD8+CD45RA-CD147++ | CD45RA vs. CD147 |
| %CD45RA-CD147+medium | CD3+CD8+ T cells | CD3+CD8+CD45RA-CD147+ | CD45RA vs. CD147 |
| %CD45RA+CD147+ | CD3+CD8+ T cells | CD3+CD8+CD45RA+CD147+ | CD45RA vs. CD147 |
| Distribution of CD39 on Tc cells ( %CD45RA-CD39+) | CD3+CD8+ T cells | CD3+CD8+CD45RA-CD39+ | CD45RA vs. CD39 |
| %CD45RA-CD39- | CD3+CD8+ T cells | CD3+CD8+CD45RA-CD39- | CD45RA vs. CD39 |
| %CD45RA+CD39+ | CD3+CD8+ T cells | CD3+CD8+CD45RA+CD39+ | CD45RA vs. CD39 |

| | | | |
|---|---|---|---|
| %CD45RA+CD39- | CD3+CD8+ T cells | CD3+CD8+CD45RA+CD39- | CD45RA vs. CD39 |
| %CD161 on CD3+CD8+ cells | CD3+CD8+ T cells | CD3+CD8+CD161+ | CD161 vs. SSC-A |
| Proliferative % CD3+CD8+ T cells | CD3+CD8+ T cells | CD3+CD8+KI67+ | Ki67 vs. SSC-A |
| %CD161 on CD3-CD8+ cells | CD3-CD8+ T cells | CD3-CD8+CD161+ | CD161 vs. SSC-A |
| **Panel 2** | | | |
| **Population** | **reported as % of** | **Phenotype** | **Gating** |
| CD3+CD4+ T cells | lymphocytes | CD3+CD4+ | CD3 vs. CD4 |
| naive CD4 | CD3+CD4+ T cells | CD3+CD4+CD45RA+CCR7+ | CD45RA vs. CCR7 |
| naive CD4 CD28+CD27+ | naive CD4 | CD3+CD4+CD45RA+CCR7+CD27+CD28+ | CD27 vs. CD28 |
| CD4 naive CD57+ PD1+ | naive CD4 CD28+CD27+ | CD3+CD4+CD45RA+CCR7+CD27+CD28+CD57+PD1+ | CD57 vs. PD1 |
| CD4 naive CD57+ PD1- | naive CD4 CD28+CD27+ | CD3+CD4+CD45RA+CCR7+CD27+CD28+CD57+PD1- | CD57 vs. PD1 |
| CD4 naive CD57- PD1+ | naive CD4 CD28+CD27+ | CD3+CD4+CD45RA+CCR7+CD27+CD28+CD57-PD1+ | CD57 vs. PD1 |
| CD4 naive CD57- PD1- | naive CD4 CD28+CD27+ | CD3+CD4+CD45RA+CCR7+CD27+CD28+CD57-PD1- | CD57 vs. PD1 |
| CD4 naive CD38+ HLADr+ | naive CD4 CD28+CD27+ | CD3+CD4+CD45RA+CCR7+CD27+CD28+CD38+HLADR+ | CD38 vs. HLADr |
| CD4 naive CD38+ HLADr- | naive CD4 CD28+CD27+ | CD3+CD4+CD45RA+CCR7+CD27+CD28+CD38+HLADR- | CD38 vs. HLADr |
| CD4 naive CD38- HLADr+ | naive CD4 CD28+CD27+ | CD3+CD4+CD45RA+CCR7+CD27+CD28+CD38-HLADR+ | CD38 vs. HLADr |

| | | | |
|---|---|---|---|
| CD4 naive CD38- HLADr- | naive CD4 CD28+CD27+ | CD3+CD4+CD45RA+CCR7+CD27+CD28+CD38-HLADR- | CD38 vs. HLADr |
| CD4 CM | CD3+CD4+ T cells | CD3+CD4+CD45RA-CCR7+ | CD45RA vs. CCR7 |
| CD4 CM CD28+CD27+ | CD4 CM | CD3+CD4+CD45RA-CCR7+CD27+CD28+ | CD27 vs. CD28 |
| CD4 CM CD57+ PD1+ | CD4 CM CD28+CD27+ | CD3+CD4+CD45RA-CCR7+CD27+CD28+CD57+PD1+ | CD57 vs. PD1 |
| CD4 CM CD57+ PD1- | CD4 CM CD28+CD27+ | CD3+CD4+CD45RA-CCR7+CD27+CD28+CD57+PD1- | CD57 vs. PD1 |
| CD4 CMCD57- PD1+ | CD4 CM CD28+CD27+ | CD3+CD4+CD45RA-CCR7+CD27+CD28+CD57-PD1+ | CD57 vs. PD1 |
| CD4 CM CD57- PD1- | CD4 CM CD28+CD27+ | CD3+CD4+CD45RA-CCR7+CD27+CD28+CD57-PD1- | CD57 vs. PD1 |
| CD4 CM CD38+ HLADr+ | CD4 CM CD28+CD27+ | CD3+CD4+CD45RA-CCR7+CD27+CD28+CD38+HLADR+ | CD38 vs. HLADr |
| CD4 CM CD38+ HLADr- | CD4 CM CD28+CD27+ | CD3+CD4+CD45RA-CCR7+CD27+CD28+CD38+HLADR- | CD38 vs. HLADr |
| CD4 CMCD38- HLADr+ | CD4 CM CD28+CD27+ | CD3+CD4+CD45RA-CCR7+CD27+CD28+CD38-HLADR+ | CD38 vs. HLADr |
| CD4 CM CD38- HLADr- | CD4 CM CD28+CD27+ | CD3+CD4+CD45RA-CCR7+CD27+CD28+CD38-HLADR- | CD38 vs. HLADr |
| CD4 EM | CD3+CD4+ T cells | CD3+CD4+CD45RA-CCR7- | CD45RA vs. CCR7 |
| CD4 EM CD27-CD28+ | CD4 EM | CD3+CD4+CD45RA-CCR7-CD27-CD28+ | CD27 vs. CD28 |
| CD4 EM CD27+CD28+ | CD4 EM | CD3+CD4+CD45RA-CCR7-CD27+CD28+ | CD27 vs. CD28 |
| CD4 EM CD27+CD28- | CD4 EM | CD3+CD4+CD45RA-CCR7-CD27+CD28- | CD27 vs. CD28 |
| CD4EM CD27-CD28- | CD4 EM | CD3+CD4+CD45RA-CCR7-CD27-CD28- | CD27 vs. CD28 |

| | | | |
|---|---|---|---|
| CD4 EM CD57+ PD1+ | CD4 EM | CD3+CD4+CD45RA-CCR7-CD57+PD1+ | CD57 vs. PD1 |
| CD4 EM CD57+ PD1- | CD4 EM | CD3+CD4+CD45RA-CCR7-CD57+PD1- | CD57 vs. PD1 |
| CD4 EM CD57- PD1+ | CD4 EM | CD3+CD4+CD45RA-CCR7-CD57-PD1+ | CD57 vs. PD1 |
| CD4 EM CD57- PD1- | CD4 EM | CD3+CD4+CD45RA-CCR7-CD57-PD1- | CD57 vs. PD1 |
| CD4 EM CD38+ HLADR+ | CD4 EM | CD3+CD4+CD45RA-CCR7-CD38+HLADR+ | CD38 vs. HLADr |
| CD4 EM CD38+ HLADR- | CD4 EM | CD3+CD4+CD45RA-CCR7-CD38+HLADR- | CD38 vs. HLADr |
| CD4 EMCD38- HLADR+ | CD4 EM | CD3+CD4+CD45RA-CCR7-CD38-HLADR+ | CD38 vs. HLADr |
| CD4 EM CD38- HLADR- | CD4 EM | CD3+CD4+CD45RA-CCR7-CD38-HLADR- | CD38 vs. HLADr |
| CD4 TEMRA | CD3+CD4+ T cells | CD3+CD4+CD45RA+CCR7- | CD45RA vs. CCR7 |
| CD4 TEMRA pE1 | CD3+CD4+ T cells | CD3+CD4+CD45RA+CCR7-CD27+CD28+ | CD27 vs. CD28 |
| CD4 TEMRA pE2 | CD3+CD4+ T cells | CD3+CD4+CD45RA+CCR7-CD27+CD28- | CD27 vs. CD28 |
| CD4 TEMRA E | CD3+CD4+ T cells | CD3+CD4+CD45RA+CCR7-CD27-CD28- | CD27 vs. CD28 |
| CD4 TEMRA CD57+ PD1+ | CD4 TEMRA | CD3+CD4+CD45RA+CCR7-CD57+PD1+ | CD57 vs. PD1 |
| CD4 TEMRA CD57+ PD1- | CD4 TEMRA | CD3+CD4+CD45RA+CCR7-CD57+PD1- | CD57 vs. PD1 |
| CD4 TEMRA CD57- PD1+ | CD4 TEMRA | CD3+CD4+CD45RA+CCR7-CD57-PD1+ | CD57 vs. PD1 |
| CD4 TEMRA CD57- PD1- | CD4 TEMRA | CD3+CD4+CD45RA+CCR7-CD57-PD1- | CD57 vs. PD1 |
| CD4 TEMRA CD38+ HLADr+ | CD4 TEMRA | CD3+CD4+CD45RA+CCR7-CD38+HLADR+ | CD38 vs. HLADr |
| CD4 TEMRA CD38+ HLADr- | CD4 TEMRA | CD3+CD4+CD45RA+CCR7-CD38+HLADR- | CD38 vs. HLADr |
| CD4 TEMRA CD38- HLADr+ | CD4 TEMRA | CD3+CD4+CD45RA+CCR7-CD38-HLADR+ | CD38 vs. HLADr |
| CD4 TEMRA CD38- HLADr- | CD4 TEMRA | CD3+CD4+CD45RA+CCR7-CD38-HLADR- | CD38 vs. HLADr |
| CD25/CD127 Tregs | CD4+ T cells | CD3+CD4+CD127low/negCD25hi | CD25 vs. CD127 |
| Treg naive | CD25/CD127 Tregs | CD3+CD4+CD127low/negCD25hiCD45RA+CCR7+ | CD45RA vs. CCR7 |
| Treg naive HLADr+ | Treg naive | CD3+CD4+CD127low/negCD25hiCD45RA+CCR7+HLADR+ | SSC-A vs. HLADR (HLADr+) |
| Treg EM | CD25/CD127 Tregs | CD3+CD4+CD127low/negCD25hiCD45RA-CCR7- | CD45RA vs. CCR7 |

| | | | |
|---|---|---|---|
| Treg EM HLADr+ | Treg EM | CD3+CD4+CD127low/negCD25hiCD45RA-CCR7-HLADR+ | Histogram HLADr (HLADr+) |
| Treg CM | CD25/CD127 Tregs | CD3+CD4+CD127low/negCD25hiCD45RA-CCR7+HLADR+ | CD45RA vs. CCR7 |
| Treg CM HLADr+ | Treg CM | CD3+CD4+CD127low/negCD25hiCD45RA-CCR7+HLADR+ | Histogram HLADr (HLADr+) |
| Treg TEMRA | CD25/CD127 Tregs | CD3+CD4+CD127low/negCD25hiCD45RA+CCR7- | CD45RA vs CCR7 |
| Treg TEMRA HLADr+ | Treg TEMRA | CD3+CD4+CD127low/negCD25hiCD45RA+CCR7-HLADR+ | Histogram HLADr (HLADr+) |
| CD279 (PD1) Tregs | CD25/CD127 Tregs | CD3+CD4+CD127low/negCD25hiCD45RA-CD279+ | CD45RA vs. CD279 (CD279+) |
| CD95 Tregs | CD25/CD127 Tregs | CD3+CD4+CD127low/negCD25hiCD45RA-CD95+ | CD45RA vs. CD95 (CD95+) |
| HLADR+ Tregs | CD25/CD127 Tregs | CD3+CD4+CD127low/negCD25hiCD45RA-HLADR+ | CD45RA vs. HLADR |
| CD45RA-CD38+ Tregs | CD25/CD127 Tregs | CD3+CD4+CD127low/negCD25hiCD45RA-CD38+ | CD45RA vs. CD38 |
| CD45RA+CD38+ Tregs | CD25/CD127 Tregs | CD3+CD4+CD127low/negCD25hiCD45RA+CD38+ | CD45RA vs. CD38 |
| CD45RA-CD38- Tregs | CD25/CD127 Tregs | CD3+CD4+CD127low/negCD25hiCD45RA-CD38- | CD45RA vs. CD38 |
| CD45RA+CD38- Tregs | CD25/CD127 Tregs | CD3+CD4+CD127low/negCD25hiCD45RA+CD38- | CD45RA vs. CD38 |
| PD1+CD95+ CD4+ | CD4+ T cells | CD3+CD4+CD279+CD95+ | PD1 vs. CD95 (PD1+, CD95+) |
| PD1+CD95- CD4+ | CD4+ T cells | CD3+CD4+CD279+CD95- | PD1 vs. CD95 (PD1+, CD95-) |
| PD1-CD95+ CD4+ | CD4+ T cells | CD3+CD4+CD279-CD95+ | PD1 vs. CD95 (PD1-, CD95+) |
| PD1-CD95- CD4+ | CD4+ T cells | CD3+CD4+CD279-CD95- | PD1 vs. CD95 (PD1-, CD95-) |

| | | | |
|---|---|---|---|
| CD57+ in CD4 T cells | CD4+ T cells | CD3+CD4+CD57+ | SSC-A vs. CD57 |
| CD45RA+CD57+ in CD4 T cells | CD4+ T cells | CD3+CD4+CD45RA+CD57+ | CD45RA vs. CD57 |
| CD45RA-CD57+ in CD4 T cells | CD4+ T cells | CD3+CD4+CD45RA-CD57+ | CD45RA vs. CD57 |
| CD45RA+CD57- in CD4 T cells | CD4+ T cells | CD3+CD4+CD45RA+CD57- | CD45RA vs. CD57 |
| CD45RA-CD57- in CD4 T cells | CD4+ T cells | CD3+CD4+CD45RA-CD57- | CD45RA vs. CD57 |
| HLADR+ in CD4 Tcells | CD4+ T cells | CD3+CD4+HLADR+ | SSC-A vs. HLADR |
| CD38+ in CD4 T cells | CD4+ T cells | CD3+CD4+CD38+ | SSC-A vs. CD38 |
| CD45RA+CD38+ in CD4 T cells | CD4+ T cells | CD3+CD4+CD45RA+CD38+ | CD45RA vs. CD38 |
| CD45RA-CD38+ in CD4 T cells | CD4+ T cells | CD3+CD4+CD45RA-CD38+ | CD45RA vs. CD38 |
| CD45RA+CD38- in CD4 T cells | CD4+ T cells | CD3+CD4+CD45RA+CD38- | CD45RA vs. CD38 |
| CD45RA-CD38- in CD4 T cells | CD4+ T cells | CD3+CD4+CD45RA-CD38- | CD45RA vs. CD38 |
| CD3+CD8+ T cells | lymphocytes | CD3+CD8+ | CD3 vs. CD8 |
| CD8 NAIVE | CD3+CD8+ T cells | CD3+CD8+CD45RA+CCR7+ | CD45RA vs. CCR7 |
| CD8 NAIVE CD27+CD28+ | CD8 naive | CD3+CD8+CD45RA+CCR7+CD27+CD28+ | CD27 vs. CD28 |
| CD8 naive CD57+ PD1+ | naive CD8 CD28+CD27+ | CD3+CD8+CD45RA+CCR7+CD27+CD28+CD57+PD1+ | CD57 vs. PD1 |
| CD8 naive CD57+ PD1- | naive CD8 CD28+CD27+ | CD3+CD8+CD45RA+CCR7+CD27+CD28+CD57+PD1- | CD57 vs. PD1 |

| | | | |
|---|---|---|---|
| CD8 naive CD57- PD1+ | naive CD8 CD28+CD27+ | CD3+CD8+CD45RA+CCR7+CD27+CD28+CD57-PD1+ | CD57 vs. PD1 |
| CD naive CD57- PD1- | naive CD8 CD28+CD27+ | CD3+CD8+CD45RA+CCR7+CD27+CD28+CD57-PD1- | CD57 vs. PD1 |
| CD8 naive CD38+ HLADR+ | naive CD8 CD28+CD27+ | CD3+CD8+CD45RA+CCR7+CD27+CD28+CD38+HLADR+ | CD38 vs. HLADr (CD38+, HLADr+) |
| CD8 naive CD38+ HLADR- | naive CD8 CD28+CD27+ | CD3+CD8+CD45RA+CCR7+CD27+CD28+CD38+HLADR- | CD38 vs. PD1 (CD57+, PD1-) |
| CD8 naive CD38- HLADR+ | naive CD8 CD28+CD27+ | CD3+CD8+CD45RA+CCR7+CD27+CD28+CD38-HLADR+ | CD38 vs. HLADr (CD38-, HLADr+) |
| CD8 naive CD38- HLADR- | naive CD8 CD28+CD27+ | CD3+CD8+CD45RA+CCR7+CD27+CD28+CD38-HLADR- | CD38 vs. HLADr (CD38-, HLADr-) |
| CD8 CM | CD3+CD8+ T cells | CD3+CD8+CD45RA-CCR7+ | CD45RA vs. CCR7 |
| CD8 CM CD27+CD28+ | CD8 CM | CD3+CD8+CD45RA-CCR7+CD27+CD28+ | CD27 vs. CD28 |
| CD8 CM CD57+ PD1+ | CD8 CM CD27+CD28+ | CD3+CD8+CD45RA-CCR7+CD27+CD28+CD57+PD1+ | CD57 vs. PD1 (CD57+, PD1+) |
| CD8 CM CD57+ PD1- | CD8 CM CD27+CD28+ | CD3+CD8+CD45RA-CCR7+CD27+CD28+CD57+PD1- | CD57 vs. PD1 (CD57+, PD1-) |
| CD8 CMCD57- PD1+ | CD8 CM CD27+CD28+ | CD3+CD8+CD45RA-CCR7+CD27+CD28+CD57-PD1+ | CD57 vs. PD1 (CD57-, PD1+) |
| CD4 CM CD57- PD1- | CD8 CM CD27+CD28+ | CD3+CD8+CD45RA-CCR7+CD27+CD28+CD57-PD1- | CD57 vs. PD1 (CD57-, PD1-) |
| CD8 CM CD38+ HLADR+ | CD8 CM CD27+CD28+ | CD3+CD8+CD45RA-CCR7+CD27+CD28+CD38+HLADR+ | CD38 vs. HLADr (CD38+, HLADr+) |

| | | | |
|---|---|---|---|
| CD8 CM CD38+ HLADR- | CD8 CM CD27+CD28+ | CD3+CD8+CD45RA-CCR7+CD27+CD28+CD38+HLADR- | CD38 vs. HLADr (CD38+, HLADr-) |
| CD8 CMCD38- HLADR+ | CD8 CM CD27+CD28+ | CD3+CD8+CD45RA-CCR7+CD27+CD28+CD38-HLADR+ | CD38 vs. HLADr (CD38-, HLADr+) |
| CD8 CM CD38- HLADR- | CD8 CM CD27+CD28+ | CD3+CD8+CD45RA-CCR7+CD27+CD28+CD38-HLADR- | CD38 vs. HLADr (CD38-, HLADr-) |
| CD8 EM | CD3+CD8+ T cells | CD3+CD8+CD45RA-CCR7- | CD45RA vs. CCR7 |
| CD8 EM CD27+CD28+ | CD8 EM | CD3+CD8+CD45RA-CCR7-CD28+CD27+ | CD27 vs. CD28 (CD28+, CD27+) |
| CD8 EM CD27-CD28+ | CD8 EM | CD3+CD8+CD45RA-CCR7-CD28-CD27+ | CD27 vs. CD28 (CD28-, CD27+) |
| CD8 EM CD27-CD28- | CD8 EM | CD3+CD8+CD45RA-CCR7-CD28-CD27- | CD27 vs. CD28 (CD28-, CD27-) |
| CD8 EM CD27+CD28- | CD8 EM | CD3+CD8+CD45RA-CCR7-CD28+CD27- | CD27 v.s CD28 (CD28+, CD27-) |
| CD8 EM CD57+ PD1+ | CD8 EM | CD3+CD8+CD45RA-CCR7-CD57+PD1+ | CD57 vs. PD1 (CD57+, PD1+) |
| CD8 EM CD57+ PD1- | CD8 EM | CD3+CD8+CD45RA-CCR7-CD57+PD1- | CD57 vs. PD1 (CD57+, PD1-) |
| CD8 EM CD57- PD1+ | CD8 EM | CD3+CD8+CD45RA-CCR7-CD57-PD1+ | CD57 vs. PD1 (CD57-, PD1+) |
| CD8 EM CD57- PD1- | CD8 EM | CD3+CD8+CD45RA-CCR7-CD57-PD1- | CD57 vs. PD1 (CD57-, PD1-) |
| CD8 EM CD38+ HLADr+ | CD8 EM | CD3+CD8+CD45RA-CCR7-CD38+HLADR+ | CD38 vs. HLADr (CD38+, HLADr+) |

| | | | |
|---|---|---|---|
| CD8 EM CD38+ HLADr- | CD8 EM | CD3+CD8+CD45RA-CCR7-CD38+HLADR- | CD38 vs. PD1 (CD57+, PD1-) |
| CD8 EMCD38- HLADr+ | CD8 EM | CD3+CD8+CD45RA-CCR7-CD38-HLADR+ | CD38 vs. HLADr (CD38-, HLADr+) |
| CD8 EM CD38- HLADr- | CD8 EM | CD3+CD8+CD45RA-CCR7-CD38-HLADR- | CD38 vs. HLADr (CD38-, HLADr-) |
| CD8 TEMRA | CD3+CD8+ T cells | CD3+CD8+CD45RA+CCR7- | CD45RA vs. CCR7 |
| CD8 TEMRA pE1 | CD3+CD8+ T cells | CD3+CD8+CD45RA+CCR7-CD27+CD28+ | CD27 vs. CD28 |
| CD8 TEMRA pE2 | CD3+CD8+ T cells | CD3+CD8+CD45RA+CCR7-CD27+CD28- | CD27 vs. CD28 |
| CD8 TEMRA E | CD3+CD8+ T cells | CD3+CD8+CD45RA+CCR7-CD27-CD28- | CD27 vs. CD28 |
| CD8 TEMRA CD57+ PD1+ | CD8 TEMRA | CD3+CD8+CD45RA+CCR7-CD57+PD1+ | CD57 vs. PD1 (CD57+, PD1+) |
| CD8 TEMRA CD57+ PD1- | CD8 TEMRA | CD3+CD8+CD45RA+CCR7-CD57+PD1- | CD57 vs. PD1 (CD57+, PD1-) |
| CD8 TEMRA CD57- PD1+ | CD8 TEMRA | CD3+CD8+CD45RA+CCR7-CD57-PD1+ | CD57 vs. PD1 (CD57-, PD1+) |
| CD8 TEMRA CD57- PD1- | CD8 TEMRA | CD3+CD8+CD45RA+CCR7-CD57-PD1- | CD57 vs. PD1 (CD57-, PD1-) |
| CD8 TEMRA CD38+ HLADr+ | CD8 TEMRA | CD3+CD8+CD45RA+CCR7-CD38+HLADR+ | CD38 vs. HLADr (CD38+, HLADr+) |
| CD8 TEMRA CD38+ HLADr- | CD8 TEMRA | CD3+CD8+CD45RA+CCR7-CD38+HLADR- | CD38 vs. PD1 (CD57+, PD1-) |
| CD8 TEMRA CD38- HLADr+ | CD8 TEMRA | CD3+CD8+CD45RA+CCR7-CD38-HLADR+ | CD38 vs. HLADr (CD38-, HLADr+) |

| | | | |
|---|---|---|---|
| CD8 TEMRA CD38- HLADr- | CD8 TEMRA | CD3+CD8+CD45RA+CCR7-CD38-HLADR- | CD38 vs. HLADr (CD38-, HLADr-) |
| CD57+ in CD8 T cells | CD8+ T cells | CD3+CD8+CD57+ | SSC-A vs. CD57 |
| CD57+CD28- CD8+ cells | CD8+ T cells | CD3+CD8+CD57+CD28- | CD57 vs. CD28 |
| CD57+CD28+CD8+ cells | CD8+ T cells | CD3+CD8+CD57+CD28+ | CD57 vs. CD28 |
| CD57+CD28- CD8+ cells | CD8+ T cells | CD3+CD8+CD57-CD28+ | CD57 vs. CD28 |
| CD57+CD28- CD8+ cells | CD8+ T cells | CD3+CD8+CD57-CD28- | CD57 vs. CD28 |
| HLADR+ in CD8 Tcells | CD8+ T cells | CD3+CD8+HLADR+ | SSC-A vs. HLADR |
| CD45RA-HLADR+ in CD8+ cells | CD8+ T cells | CD3+CD8+CD45RA-HLADR+ | CD45RA vs. HLADR |
| CD45RA+HLADR+ in CD8+ cells | CD8+ T cells | CD3+CD8+CD45RA+HLADR+ | CD45RA vs. HLADR |
| CD45RA+HLADR- in CD8+ cells | CD8+ T cells | CD3+CD8+CD45RA+HLADR- | CD45RA vs. HLADR |
| CD45RA-HLADR- in CD8+ cells | CD8+ T cells | CD3+CD8+CD45RA-HLADR- | CD45RA vs. HLADR |
| PD-1+CD95+ CD8+ | CD8+ T cells | CD3+CD8+CD279+CD95+ | PD1 vs. CD95 (PD1+, CD95+) |
| PD-1+CD95- CD8+ | CD8+ T cells | CD3+CD8+CD279+CD95- | PD1 vs. CD95 (PD1+, CD95-) |
| PD-1-CD95+ CD8+ | CD8+ T cells | CD3+CD8+CD279-CD95+ | PD1 vs. CD95 (PD1-, CD95+) |
| PD-1-CD95- CD8+ | CD8+ T cells | CD3+CD8+CD279-CD95- | PD1 vs. CD95 (PD1-, CD95-) |

| | | | |
|---|---|---|---|
| CD45RA+CD38+ in CD8 T cells | CD8+ T cells | CD3+CD8+CD45RA+CD38+ | CD45RA vs. CD38 |
| CD45RA-CD38+ in CD8 T cells | CD8+ T cells | CD3+CD8+CD45RA-CD38+ | CD45RA vs. CD38 |
| CD45RA+CD38- in CD8 T cells | CD8+ T cells | CD3+CD8+CD45RA+CD38- | CD45RA vs. CD38 |
| CD45RA-CD38- in CD8 T cells | CD8+ T cells | CD3+CD8+CD45RA-CD38- | CD45RA vs. CD38 |
| **Panel 3** | | | |
| **Population** | **reported as % of** | **Phenotype** | **Gating** |
| % CD19+ in lymphocytes | lymphocytes | CD19pos | CD19 vs. SSC-A/CD19 Histogramm |
| % CD19+CD20+ B cells in lymphocytes | lymphocytes | CD19+CD20+ | CD20 vs. CD19 |
| % activated B cells in B cells | CD19+CD20+ B cells | CD19+CD20+CD86+ | SSC-A vs. CD86 |
| % CCR7+ B cells in B cells | CD19+CD20+ B cells | CD19+CD20+CCR7+ | SSC-A vs. CCR7 |
| % B1-type B cells in B cells | CD19+CD20+ B cells | CD19+CD20+CD27-CD5+ | CD27 vs. CD5 |
| % activated B1-type B cells in B1-type B cells | B1-type B cells | CD19+CD20+CD27-CD5+CD86+ | SSC-A vs. CD86 |
| % CCR7+ B1-type B cells in B1-type B cells | B1-type B cells | CD19+CD20+CD27-CD5+CCR7+ | SSC-A vs. CCR7 |
| % IgD post-switched B cells in B cells | CD19+CD20+ B cells | CD19+CD20+IgD-CD27+ | IgD vs. CD27 |

| | | | |
|---|---|---|---|
| % activated IgD post-switched B cells in IgD post-switched B cells | IgD post-switched B cells | CD19+CD20+IgD-CD27+CD86+ | SSC-A vs. CD86 |
| % CCR7+ IgD post-switched B cells in IgD post-switched B cells | IgD post-switched B cells | CD19+CD20+IgD-CD27+CCR7+ | SSC-A vs. CCR7 |
| % IgD-CD27- B cells in B cells | CD19+CD20+ B cells | CD19+CD20+IgD-CD27- | IgD vs. CD27 |
| % activated IgD-CD27- B cells in IgD-CD27- B cells | IgD-CD27- B cells | CD19+CD20+IgD-CD27-CD86+ | SSC-A vs. CD86 |
| % CCR7+ IgD-CD27- B cells in IgD-CD27- B cells | IgD-CD27- B cells | CD19+CD20+IgD-CD27-CCR7+ | SSC-A vs. CCR7 |
| % marginal zone B cells in B cells | CD19+CD20+ B cells | CD19+CD20+IgD+CD27+ | IgD vs. CD27 |
| % activated marginal zone B cells in marginal zone B cells | marginal zone B cells | CD19+CD20+IgD+CD27+CD86+ | SSC-A vs. CD86 |
| % CCR7+ marginal zone B cells in marginal zone B cells | marginal zone B cells | CD19+CD20+IgD+CD27+CCR7+ | SSC-A vs. CCR7 |
| % naive B cells in B cells | CD19+CD20+ B cells | CD19+CD20+IgD+CD27- | IgD vs. CD27 |
| % activated naive B cells in naive B cells | naive B cells | CD19+CD20+IgD+CD27-CD86+ | SSC-A vs. CD86 |
| % CCR7+ naive B cells in naive B cells | naive B cells | CD19+CD20+IgD+CD27-CCR7+ | SSC-A vs. CCR7 |

| | | | |
|---|---|---|---|
| % CD27+ B cells in B cells | CD19+CD20+ B cells | CD19+CD20+CD27+ | SSC-A vs. CD27 |
| % activated CD27+ B cells in CD27+ B cells | CD27+ B cells | CD19+CD20+CD27+CD86+ | SSC-A vs. CD86 |
| % CCR7+ CD27+ B cells in CD27+ B cells | CD27+ B cells | CD19+CD20+CD27+CCR7+ | SSC-A vs. CCR7 |
| % Class-switched memory B cells in B cells | CD27+ B cells | CD19+CD20+CD27+IgM-IgD- | IgM vs. IgD |
| % activated Class-switched memory B cells in Class-switched memory B cells | Class-switched memory B cells | CD19+CD20+CD27+IgM-IgD-CD86+ | SSC-A vs. CD86 |
| % CCR7+ Class-switched memory B cells in Class-switched memory B cells | Class-switched memory B cells | CD19+CD20+CD27+IgM-IgD-CCR7+ | SSC-A vs. CCR7 |
| % IgM only memory B cells in B cells | CD27+ B cells | CD19+CD20+CD27+IgM+IgD- | IgM vs. IgD |
| % activated IgM only memory B cells in IgM only memory B cells | IgM only memory B cells | CD19+CD20+CD27+IgM+IgD-CD86+ | SSC-A vs. CD86 |
| % CCR7+ IgM only memory B cells in IgM only memory B cells | IgM only memory B cells | CD19+CD20+CD27+IgM+IgD-CCR7+ | SSC-A vs. CCR7 |
| % IgM-IgD+ memory B cells in B cells | CD27+ B cells | CD19+CD20+CD27+IgM-IgD+ | IgM vs. IgD |

| | | | |
|---|---|---|---|
| % activated IgM-IgD+ memory B cells in IgM-IgD+ memory B cells | IgM-IgD+ memory B cells | CD19+CD20+CD27+IgM-IgD+CD86 | SSC-A vs. CD86 |
| % CCR7+ IgM-IgD+ memory B cells in IgM-IgD+ memory B cells | IgM-IgD+ memory B cells | CD19+CD20+CD27+IgM-IgD+CCR7 | SSC-A vs. CCR7 |
| % Non-switched memory B cells in B cells | CD27+ B cells | CD19+CD20+CD27+IgM+IgD+ | IgM vs. IgD |
| % activated Non-switched memory B cells in Non-switched memory B cells | Non-switched memory B cells | CD19+CD20+CD27+IgM+IgD+CD86 | SSC-A vs. CD86 |
| % CCR7+ Non-switched memory B cells in Non-switched memory B cells | Non-switched memory B cells | CD19+CD20+CD27+IgM+IgD+CCR7 | SSC-A vs. CCR7 |
| % CD27highCD38high ASC in B cells | CD19+CD20+ B cells | CD19+CD20+CD27hiCD38hi | CD27 vs. CD38 |
| % activated CD27highCD38high ASC in CD27highCD38high ASC | CD27highCD38high ASC | CD19+CD20+CD27hiCD38hiCD86+ | SSC-A vs. CD86 |
| % CCR7+ CD27highCD38high ASC in CD27highCD38high ASC | CD27highCD38high ASC | CD19+CD20+CD27hiCD38hiCCR7+ | SSC-A vs. CCR7 |
| % OMIP CD10+ B cells in B cells | CD19+CD20+ B cells | CD19+CD20+CD10+ | FSC-A vs. CD10 |

| | | | |
|---|---|---|---|
| % CCR7+ OMIP CD10+ B cells in OMIP CD10+ B cells | CD10+ B cells | CD19+CD20+CD10+CCR7+ | SSC-A vs. CCR7 |
| % OMIP Transitional B cells in OMIP CD10+ B cells | CD10+ B cells | CD19+CD20+CD10+IgD+CD38+ | IgD vs. CD38 |
| % OMIP Transitional B cells in B cells | CD19+CD20+ B cells | CD19+CD20+CD10+IgD+CD38+ | IgD vs. CD38 |
| % CCR7+ OMIP Transitional B cells in OMIP Transitional B cells | CD10+transitional B cells | CD19+CD20+CD10+IgD+CD38+CCR7+ | SSC-A vs. CCR7 |
| % OMIP CD10- B cells in B cells | CD19+CD20+ B cells | CD19+CD20+CD10- | FSC-A vs. CD10 |
| % CCR7+ OMIP CD10- B cells in OMIP CD10- B cells | CD10- B cells | CD19+CD20+CD10-CCR7+ | SSC-A vs. CCR7 |
| % OMIP unswitched B cells in OMIP CD10- B cells | CD10- B cells | CD19+CD20+CD10-IgD+CD38- | IgD vs. CD38 |
| % OMIP unswitched B cells in B cells | CD19+CD20+ B cells | CD19+CD20+CD10-IgD+CD38- | IgD vs. CD38 |
| % CCR7+ OMIP unswitched B cells in OMIP unswitched B cells | OMIP unswitched B cells | CD19+CD20+CD10-IgD+CD38-CCR7+ | SSC-A vs. CCR7 |
| % OMIP marginal Zone B cells in OMIP unswitched B cells | OMIP unswitched B cells | CD19+CD20+CD10-IgD+CD38-CD27+ | IgD vs. CD27 |
| % CCR7+ OMIP marginal Zone B cells in OMIP marginal Zone B cells | OMIP marginal Zone B cells | CD19+CD20+CD10-IgD+CD38-CD27+CCR7+ | SSC-A vs. CCR7 |

| | | | |
|---|---|---|---|
| % OMIP naive B cells in OMIP unswitched B cells | OMIP unswitched B cells | CD19+CD20+CD10-IgD+CD38-CD27- | IgD vs. CD27 |
| % CCR7+ OMIP naive B cells in OMIP naive B cells | OMIP naive B cells | CD19+CD20+CD10-IgD+CD38-CD27-CCR7+ | SSC-A vs. CCR7 |
| % OMIP Plasmablasts in OMIP CD10- B cells | OMIP CD10- B cells | CD19+CD20+CD10-IgD-CD38+ | IgD vs. CD38 |
| % OMIP Plasmablasts in B cells | CD19+CD20+ B cells | CD19+CD20+CD10-IgD-CD38+ | IgD vs. CD38 |
| % CCR7+ OMIP Plasmablasts in OMIP Plasmablasts | OMIP Plasmablasts | CD19+CD20+CD10-IgD-CD38+CCR7+ | SSC-A vs. CCR7 |
| % OMIP Memory B cells in OMIP CD10- B cells | OMIP CD10- B cells | CD19+CD20+CD10-IgD-CD38- | IgD vs. CD38 |
| % OMIP Memory B cells in B cells | CD19+CD20+ B cells | CD19+CD20+CD10-IgD-CD38- | IgD vs. CD38 |
| % CCR7+ OMIP Memory B cells in OMIP Memory B cells | OMIP memory B cells | CD19+CD20+CD10-IgD-CD38-CCR7+ | SSC-A vs. CCR7 |
| % OMIP resting Memory B cells in OMIP Memory B cells | OMIP memory B cells | CD19+CD20+CD10-IgD-CD38-CD21+CD27+ | CD21 vs. CD27 |
| % CCR7+ OMIP resting Memory B cells in OMIP resting Memory B cells | OMIP resting memory B cells | CD19+CD20+CD10-IgD-CD38-CD21+CD27+CCR7+ | SSC-A vs. CCR7 |
| % OMIP intermediate Memory B cells in OMIP Memory B cells | OMIP memory B cells | CD19+CD20+CD10-IgD-CD38-CD21+CD27- | CD21 vs. CD27 |

| | | | |
|---|---|---|---|
| % CCR7+ OMIP intermediate Memory B cells in OMIP intermediate Memory B cells | OMIP intermediate memory B cells | CD19+CD20+CD10-IgD-CD38-CD21+CD27-CCR7+ | SSC-A vs. CCR7 |
| % OMIP activated Memory B cells in OMIP Memory B cells | OMIP memory B cells | CD19+CD20+CD10-IgD-CD38-CD21-CD27+ | CD21 vs. CD27 |
| % CCR7+ OMIP activated Memory B cells in OMIP activated Memory B cells | OMIP activated memory B cells | CD19+CD20+CD10-IgD-CD38-CD21-CD27+CCR7+ | SSC-A vs. CCR7 |
| % OMIP exhausted tissue-like Memory B cells in OMIP Memory B cells | OMIP memory B cells | CD19+CD20+CD10-IgD-CD38-CD21-CD27- | CD21 vs. CD27 |
| % CCR7+ OMIP exhausted tissue-like Memory B cells in OMIP exhausted tissue-like Memory B cells | OMIP exhausted tissue like memory B cells | CD19+CD20+CD10-IgD-CD38-CD21-CD27-CCR7+ | SSC-A vs. CCR7 |
